# The causal relationships between obstructive sleep apnea and elevated CRP and TNF-α protein levels

**DOI:** 10.1080/07853890.2022.2081873

**Published:** 2022-06-02

**Authors:** Minhan Yi, Wangcheng Zhao, Yun Tan, Quanming Fei, Kun Liu, Ziliang Chen, Yuan Zhang

**Affiliations:** a Department of Respiratory Medicine, Xiangya Hospital, Central South University, Changsha, China; b School of Life Sciences, Central South University, Changsha, China; c National Clinical Research Center for Geriatric Disorders, Xiangya Hospital, Central South University, Changsha, China; d Xiangya School of Medicine, Central South University, Changsha, China; e School of Computer Science and Engineering, Central South University, Changsha, China

**Keywords:** Obstructive sleep apnea, CRP, TNF-α, Mendelian Randomization, CPAP

## Abstract

**Background:**

Obstructive sleep apnea (OSA) and inflammation are closely related. This study aimed to evaluate the associations and causal effect between C-reactive protein (CRP) and tumour necrosis factor-alpha (TNF-α) levels and OSA.

**Methods:**

Pooled analysis was conducted to compare the expression differences of CRP and TNF-α between OSA patients with different severity and controls, and between continuous positive airway pressure (CPAP) and non-CPAP interventions for OSA patients. Using published GWAS summary statistics, we conducted a bidirectional two-sample Mendelian Randomization (MR) to estimate the causal relationships between CRP and TNF-α levels and OSA risk. Effect estimates were evaluated using inverse-variance weighted (IVW) as primary method, and several other MR methods as sensitivity analysis.

**Results:**

Both TNF-α (WMD [95%CI] = 5.86 [4.80–6.93] pg/ml, *p* < .00001) and CRP (WMD [95%CI] = 2.66 [2.15–3.17] mg/L, *p* < .00001), showed a significant increase in OSA patients compared with controls and this increasing trend was associated with OSA severity. Besides, compared to blank control (non-CPAP), CPAP treatment can reduce high TNF-α (WMD [95%CI]= −4.44 [−4.81, −4.07]pg/ml, *p* < .00001) and CRP (WMD [95%CI]= −0.91 [−1.65, −0.17] mg/l, *p* = .02) in OSA. Moreover, the primary MR analysis by IVW showed that OSA was the genetically predicted cause of elevated CRP (estimate: 0.095; 95% CI, [0.010–0.179]; *p* = .029) using six SNPs as the instrument variable, which were repeated by weighted median (estimate: 0.053; 95% CI, [0.007, 0.100]; *p* =.024) and MR RAPS (estimate: 0.109; 95% CI, [0.079, 0.140]; *p* = 1.98x10^−12^). Besides, the causal effect from elevated CRP on increased OSA risk was almost significant by IVW (OR:1.053; 95% CI, [1.000, 1.111]; *p* = .053). However, there were no causal associations between TNF-α and OSA from both directions.

**Conclusions:**

Increased CRP and TNF-α were associated with OSA severity and sensible to CPAP treatment. Also, OSA had a suggestive causal effect on elevated CRP.

## Introduction

Obstructive sleep apnea (OSA) is a widespread sleep disorder that affects about 10–25% of the global population [1,2]. It is characterized by recurrent episodes of constriction or collapse of the upper airway during sleep, which can lead to intermittent hypoxia (IH), sympathetic overactivity, sleep fragmentation and disarrangement to physiological homoeostasis [3]. During these processes, systematic inflammation has been reported to play a critical role. Activated inflammation pathways and changed inflammation protein levels can either be a cause or consequence of OSA [4]. Understanding the association and causality between key inflammation proteins and OSA is helpful in studying the mechanism and developing new treatments for OSA.

Common biomarkers serving as indicators of systemic inflammation include CRP and TNF-α^5^. CRP and TNF-α have been widely studied in OSA and have shown to be elevated in OSA patients in several epidemiological studies [6–8], while the results in some studies were inconsistent [9–11]. Besides, the associations between OSA-severity and the levels of CRP and TNF-α are still unclear [12–14]. But these studies were limited to a relatively small sample and the data need to be updated. Thus, a pooled analysis depending on the larger sample size is required to assess the associations between CRP and TNF-α levels and OSA. Also, the impact of continuous positive airway pressure (CPAP), an effective therapy for OSA [15], on CRP and TNF-α levels is important for exploring the link between OSA and inflammation. If the hypothesis that OSA causes inflammation is indeed correct, it is theoretically possible that the alleviation of OSA may be accompanied by a decrease in inflammation proteins.

The majority of previous studies on OSA have only shown an inflammatory profile in patients with OSA; it is unclear whether the altered inflammatory profile occurs before or after the onset of OSA, or both. Mendelian Randomization (MR) is a reliable genetic epidemiology method that uses genetic variants as instrumental variable (IV) to assure whether causality exists between OSA and inflammation [16]. Because the genetic variants are naturally randomized to offspring during conception, MR can avoid the influence of confounding and reverse causation which was the common limitation in observational studies [17,18]. Currently, no MR analysis has examined the causality between inflammation and OSA risk. Multiple genetic loci were identified for OSA, CRP and TNF-α in recently published genomewide association studies (GWAS), and the GWAS summary statistics for OSA (217,955 participants), CRP (341,805 individuals) and TNF-α (3454 individuals) are available [19–21], making it possible to conduct a powerful MR analysis.

In this study, we first explored the association between the levels of CRP, TNF-α and OSA severity, and whether this aberration can be reduced by CPAP treatment. Then, we applied a bidirectional MR analysis to estimate the causal relationships between these two cytokines and OSA risk.

## Materials and methods

### Meta-analysis of associations between CRP and TNF-α levels and OSA

To compare the expression differences of TNF-α and CRP between OSA patients with different severity and controls, and between CPAP treatment and non-CPAP intervention for OSA patients, studies were identified from published literature. Detailed descriptions of searching strategy, inclusive and exclusive criteria and data extraction were presented in Supplementary Methods.

All data were analysed in the Review manager 5.3 (The Nordic Cochrane Centre, The Cochrane Collaboration, London, UK). The measurement units of CRP and TNF-α were standardised. For CRP, mg/dl was converted to mg/l, TNF-α was pg/ml. As for continuous outcomes, the weighted mean differences (WMD) and 95% confidence interval (CI) were used as measures of the effect between the two groups in this study. We calculate an I^2^ statistic to estimate heterogeneity. If I^2^ > 50%, the data were pooled by random effect model, otherwise by fixed effect model. We also performed a sensitivity analysis by removing article one by one to see its effect on the *p* value. Moreover, a funnel diagram was conducted to evaluate publication bias^22^.

### Mendelian Randomization (MR) analysis investing causal relationship

#### GWAS summary statistics sources

A two-sample MR model was used to evaluate the causal effect between OSA and protein levels of CRP and TNF-α respectively. The GWAS summary statistics for OSA were from a recent publication using FinnGen Study which contained 217,955 individuals with 16,761 OSA patients from European [20]. In this GWAS, OSA was diagnosed based on the International Statistical Classification of Diseases (ICD) codes (ICD-10: G47.3, ICD-9: 3472 A). Besides, the participants of CRP GWAS summary statistics were from the UK Biobank, including 341,805 individuals from European [19]. The UK Biobank is a prospective cohort that recruited over half a million men and women aged 40–96 between 2006 and 2010 and tracked their health over time [23]. TNF-α GWAS summary statistics were identified from a GWAS using YFS and FINRISK2002 including 3454 individuals from Finland [21]. And the whole TNF-α was assessed by ELISA [21].

#### Instrumental variable selection

There are three assumptions for instrumental variable (IV) selection in two-sample MR analysis: (1) the selected variants are associated with exposure; (2) the IV variants are not associated with the confounding factors of exposure and outcome; (3) the effect from IV variants to outcome was exclusively through exposure [24]. In detail, when using CRP and OSA as exposure, we selected associated variants with *p* < 5 × 10^−8^ [19]. Because of the limitation of sample size, the SNPs associated with TNF-α were selected with *p* < 5 × 10^−6^ [21]. Then, clumping was performed with criteria of distance = 1000kb and r^2^=0.1 to include independently associated variants as IV. After harmonising with responsive outcome, each pair was obtained for subsequent analysis. Besides, F-statistic was used to assess the strength of the association between SNP and exposure, where score > 10 indicates that the instrument is sufficiently strong [25].

#### Data analysis

We used several two-sample MR approaches to evaluate the causal effect between OSA and CRP, also OSA and TNF-α. First, we select SNPs of OSA as exposure to evaluate the causal effect of OSA on CRP and TNF-α. Then, the SNPs of OSA were performed as the outcome to assess the causal effect of CRP and TNF-α on OSA.

We used inverse-variance weighted (IVW) as the main outcome which assumes that all SNPs are valid instruments [26]. In sensitivity analyses, we used MR Egger [27], Weighted median [28] and MR-Robust Adjusted Profile Score (RAPS) [29] to correct for any potential violations of the assumptions. We used these three analyses as they operate in different ways and rely on different assumptions for valid inferences to assess the reliability of MR analyses. In addition, heterogeneity was analysed by Cochran’s Q test of IVW and MR Egger, pleiotropy was tested by the intercept of MR Egger analysis. When heterogeneity was detected for associated relationships, we used the RadialMR package to remove outliers and performed above analysis again [30].

## Results

### Protein levels of TNF-α and CRP in OSA

**Figure 1.**
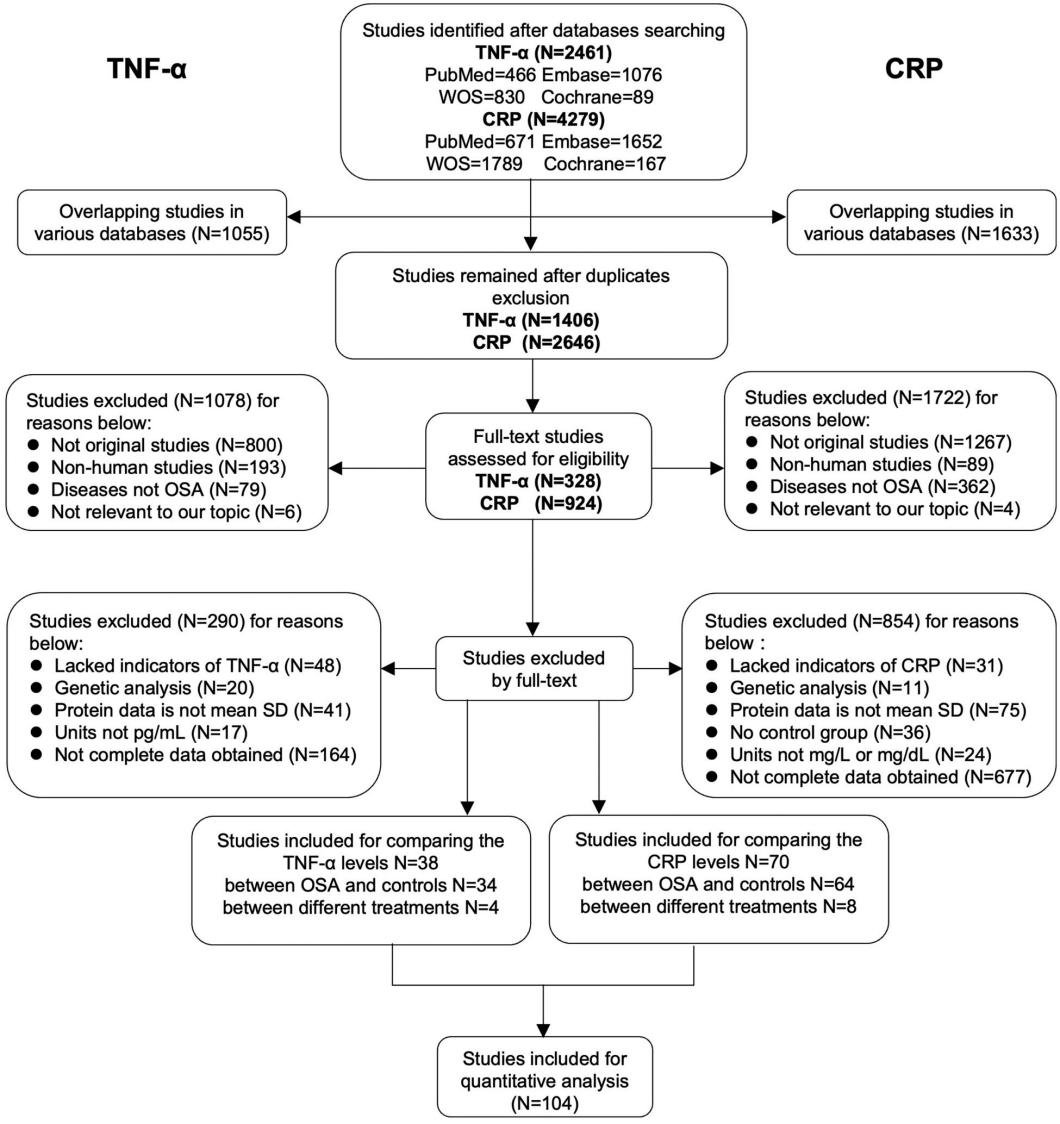
Flow diagram of literature selecting based on the inclusion and exclusion criteria. The left and right parts of the flow diagram represent the retrieval and screening process of TNF-α and CRP respectively. There are several articles that provided both comparison data between OSA and controls and between different treatments in OSA participants, or even one paper covered both TNF-α and CRP, thus the total count of included papers were less than the direct sum of each part.

To compare the association between TNF-α and CRP levels and OSA patients, a total of 104 studies were included in the pooled qualitative analysis (Figure 1), and the NOS score of every study ranged from 4 to 9. The extracted original details are presented in supplementary tables.

#### Elevated CRP and TNF-α levels in OSA are related to its severity

**Figure 2.**
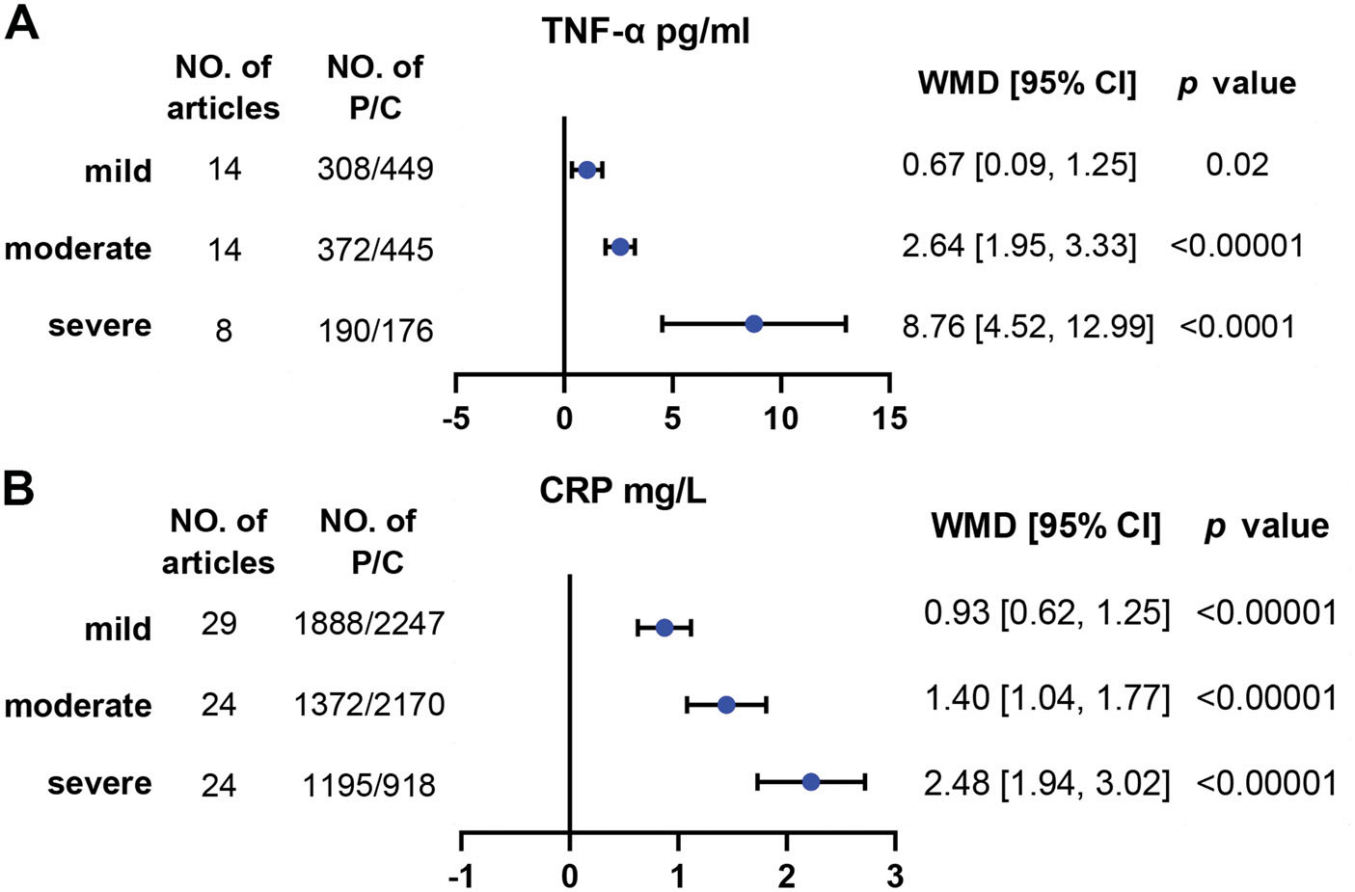
Inflammation proteins of TNF-α and CRP were associated with severity of OSA. Weighted mean difference (WMD) and 95% confidence interval (95%CI) for inflammation proteins levels of TNF-α (A), CRP (B) were compared between mild, moderate, severe OSA patients and controls respectively. NO. of articles: number of articles included for analysis in each group. NO. of P/C: number of OSA patients (P) and controls (C) were included for each group. The statistically different results with *p*<.05 were shown in blue point.

Firstly, we compared the level of TNF-α between 1981 OSA patients and 1112 controls in 34 studies and the level of CRP between 4285 OSA patients and 3692 controls in 64 studies (Supplemental Tables 1–4). We found both TNF-α (WMD [95%CI] = 5.86 [4.80–6.93] pg/ml, *p* < .00001) and CRP (WMD [95%CI] = 2.66 [2.15–3.17] mg/L, *p* < .00001) showed a significant increase in OSA patients compared with controls (Tables 1–2, Supplemental Figures 1–2). Besides, there were increasing trends with statistical significance from mild to moderate and then to severe for TNF-α and CRP (Figure 2, Supplemental Figures 3–4). Sensitivity analysis suggested significantly higher trends of TNF-α and CRP in OSA. The funnel plot results indicated no apparent publication bias (Supplemental Figure 5).

**Table 1.** Summary of studies included in analysis of TNF-α levels between OSA and controls.

Study ID	Region	PMID	P/C NO.	TNF-α pg/ml		NOS
				Case	Control	
Bilal N. 2021	Turkey	32776303	30/30	16.11 ± 2.2*	10.58 ± 1.71	8
Ji L. 2021	China	32572684	67/21	138.9 ± 105.3*	34.9 ± 21.9	8
Chen V.G. 2020	Brazil	30213594	17/17	50.39 ± 25.86	44.41 ± 13.77	5
Chuang H. H. 2020	Taiwan	32093397	11/24	44.9 ± 19.3	45.9 ± 19.2	6
Ming H. 2019	China	30783447	684/192	31.2 ± 5.3	12.1 ± 1.1	8
Rogers V.E. 2018	US	29862666	20/7	4.5 ± 3.18	5.24 ± 3.25	5
Bozic J. 2018	Croatia	29991422	50/25	8.67 ± 2.41*	2.35 ± 1.25	8
Gamsiz-Isik H. 2017	Turkey	27858556	83/80	11.5 ± 3.11	11.25 ± 4	8
Jin F. 2017	China	28901415	100/50	37.67 ± 0.21	29.15 ± 1.74	8
Smith D.F. 2017	US	28204724	65/90	6 ± 4.1*	10.1 ± 45.4	5
Zhang Z. 2017	China	28367199	50/52	3.01 ± 0.21	2.89 ± 0.23	5
De Santis S. 2015	Italy	26224223	26/24	122.2 ± 12	80.2 ± 18.3	8
Leon-Cabrera S. 2015	Mexico	25944984	29/13	337.9 ± 67.8	306.9 ± 38.9	6
Ciccone M.M. 2014	Italy	24481114	80/40	20.09 ± 5.39	12.53 ± 3.48	7
Nobili V. 2014	Italy	24256086	39/26	2.2 ± 6.6	6.8 ± 2	8
Unuvar Dogan F. 2014	Turkey	24895539	33/24	0.128 ± 0.15	0.108 ± 0.027	7
Akinnusi M. 2013	US	23239459	25/18	6.83 ± 2.55	1.57 ± 0.44	8
Alexopoulos E.I. 2013	Greece	24179295	46/22	0.63 ± 0.2*	0.63 ± 0.17	8
Yang D. 2013	China	23567762	25/25	12.55 ± 8.09	5.12 ± 1.23	7
Medeiros C. A. M. 2012	Brazil	21916851	50/15	2.09 ± 7.3*	0.32 ± 0.77	7
Kim J. 2010	Korea	20855682	37/22	15.32 ± 6.8*	14.4 ± 4.13	6
Sahlman J. 2010	Finland	20040038	84/40	1.54 ± 1.75	1.17 ± 1.58	7
Steiropoulos P. 2010	Greece	20628509	38/23	6.72 ± 3.72	3.94 ± 1.34	7
Li Y. 2009	China	18207457	68/22	132.1 ± 10.8*	87.3 ± 6.1	7
Antonopoulou S. 2008	Greece	18606530	45/25	1.4 ± 0.9	0.64 ± 0.3	7
Constantinidis J. 2008	Greece	18317790	13/12	124.64 ± 96.7	78.8 ± 50.1	7
Kanbay A. 2008	Turkey	18487876	106/32	114.15 ± 144.15	34.25 ± 13.1	7
Tomiyama H. 2008	Japan	18224268	50/15	2.2 ± 0.6*	1.7 ± 0.5	8
Chen J. 2007	China	–	100/40	127.6 ± 37.76	93.13 ± 32.14	6
Kobayashi K. 2006	Japan	16537861	35/16	1.11 ± 0.46	0.62 ± 0.44	6
Ciftci T. U. 2004	Turkey	15381186	43/22	4.6 ± 3.39	3.29 ± 2.13	6
Minoguchi K. 2004	Japan	15539715	24/12	2.34 ± 0.54*	1.12 ± 0.39	6
Alberti A. 2003	Italy	14633242	18/20	26.9 ± 3.45	6.5 ± 1.55	7
Liu H. 2000	China	11215046	22/16	299.09 ± 43.57	101.88 ± 21.27	6

P/C NO.: number of OSA patients (P) and controls (C) in each group. TNF-α (pg/ml) level was expressed as mean ± standard deviation (SD). NOS represents Newcastle-Ottawa Scale which was used for article quality evaluation.

*: the presented TNF-α protein level in OSA patients for this paper was from severe group because the paper provides protein data of different severity.

Detailed information for each included article, please refer to Supplement Table 1.

Detailed bibliography for each included publication, please refer to Supplement Table 2.

**Table 2.** Summary of studies included in analysis of CRP levels between OSA and controls.

Study ID	Region	PMID	P/C NO.	CRP mg/L		NOS
				Case	Control	
Bhatt S. P. 2021	India	34086720	190/57	3.7 ± 2.12	1.5 ± 0.71	7
Chen Y. C. 2021	Taiwan	34667186	56/16	3.64 ± 4.73	1.79 ± 1.11	7
Cignarelli A. 2021	Italy	34173961	68/22	82 ± 72	66 ± 71	6
Cilekar S. 2021	Turkey	34764367	70/40	31.2 ± 31.5*	21.0 ± 12.1	7
Jung J.H. 2021	Korea	31646893	87/21	0.6 ± 0.74*	0.38 ± 0.33	7
Pelaia C. 2021	Italy	34434938	371/40	48 ± 38*	25 ± 13	7
Perticone M. 2021	Italy	33748160	65/100	6.5 ± 6.4	3.5 ± 2.4	8
Rong W. 2021	China	–	42/42	7.02 ± 0.34	2.88 ± 0.22	6
Wang L.J. 2021	China	33613657	122/130	52.81 ± 27	23.99 ± 10.91	6
Azar C. 2020	Turkey	32718134	99/50	9.3 ± 6.5	9.3 ± 9.3	6
Brener A. 2020	Israel	32814798	14/44	6.4 ± 2.3	4.3 ± 2.2	6
Chen D.D. 2020	China	31473914	73/17	1.22 ± 0.34*	0.81 ± 0.22	8
Chien M.Y. 2020	China	32095087	20/20	2.3 ± 1.4	1.2 ± 0.6	8
Chu A.A. 2020	China	33214634	71/31	5.4 ± 1.1	2.0 ± 0.5	7
Huang Y.S. 2020	China	32260590	55/32	3.37 ± 6.04	0.42 ± 0.23	9
Morell-Garcia D. 2020	Spain	33043053	99/76	6 ± 16*	2 ± 2	7
Sanz-Rubio D. 2020	Spain	32210181	74/31	4 ± 3.4*	1.3 ± 1.2	8
Xie J.Y. 2020	China	32141573	107/34	3.14 ± 2.77*	0.82 ± 1.21	6
Zhang L. 2020	China	32002742	134/19	10.8 ± 5.6*	8 ± 5.4	6
Bauça J.M. 2019	Spain	30928393	209/152	41.34 ± 60.87	36.08 ± 61.19	5
Bhatt S.P. 2019	India	30032465	47/25	3.6 ± 1.5	1.4 ± 0.7	6
Voulgaris A. 2019	Greece	29946947	64/32	5.5 ± 5.8	3.4 ± 3.6	8
Aydin S. 2018	Turkey	29475915	47/17	42 ± 38	20 ± 21	6
Horvath P. 2018	Hungary	29493039	50/26	4.2 ± 3.7	4 ± 1.8	5
Kunos L. 2018	Hungary	29740686	45/31	6.3 ± 13	2.8 ± 2.4	5
Mônico-Neto M. 2018	Brazil	30093572	447/211	3.4 ± 0.4*	2.9 ± 0.5	4
Zhang D.M. 2018	China	30151379	30/20	2.09 ± 1.8	1.19 ± 1.14	7
Alonso-Álvarez M.L. 2017	Spain	28899517	62/51	4.71 ± 10.95	2.54 ± 2.60	7
Gamsiz-Isik H. 2017	Turkey	27858556	83/80	4.95 ± 8.95	2.38 ± 1.80	8
Jin F. 2017	China	28901415	100/50	49.64 ± 21.66	6.37 ± 1.30	8
Liu C.D. 2017	China	–	97/30	7.5 ± 3.5	1.6 ± 0.7	7
Masood R.K. 2017	Pakistan	–	217/63	1.72 ± 1.47*	0.63 ± 0.35	4
Nakabayashi K. 2017	Japan	28631078	134/109	0.96 ± 2.49	0.52 ± 1.41	8
Pusuroglu H. 2017	Turkey	28150280	87/21	52 ± 59	36 ± 41	8
Song T.J. 2017	China	28168435	189/94	9 ± 28*	6 ± 15	8
Xu Q. 2017	China	28966342	33/30	1.47 ± 1.6	0.97 ± 1.22	8
Zhang D. 2017	China	29301595	38/10	2.19 ± 1.94	2.09 ± 3.46	6
Cao Z. 2016	China	27228976	192/56	14.2 ± 3.3*	9.2 ± 1.2	6
Huang Y.S. 2016	China	27741107	47/32	1.90 ± 0.44	0.41 ± 0.48	6
Kim J. 2016	Korea	27684378	862/973	14.7 ± 16*	9.7 ± 12.2	7
Li F. 2016	China	26858795	153/35	6.45 ± 7.25*	2.33 ± 2.82	8
Tanriverdi H. 2016	Turkey	–	53/24	2.8 ± 3.8	2.05 ± 3.38	4
Uygur F. 2016	Turkey	26810494	96/31	3.6 ± 1.8	1.4 ± 0.9	7
Zhang H. 2016	China	–	41/19	4.25 ± 0.61	3.32 ± 0.35	6
Wang Y. 2015	China	26298789	47/28	8.4 ± 0.7	8.1 ± 0.8	6
Ciccone M.M. 2014	Italy	24481114	80/40	1.84 ± 0.67*	1.08 ± 0.53	7
Fouda N. 2014	Egypt	/	14/16	6.7 ± 0.6	4.9 ± 0.3	7
Van Eyck A. 2014	Belgium	23999834	35/85	1.95 ± 3.63*	1.8 ± 3.6	8
Yardim-Akaydin S. 2014	Turkey	23862972	139/27	62.3 ± 54.7	33.2 ± 4.9	7
Bezerra P.C 2013	Brazil	23183853	27/21	6.7 ± 5	8 ± 7.9	7
Israel L.P. 2013	Israel	24293770	25/24	4.5 ± 2.1	1.5 ± 1	8
Nural S. 2013	Turkey	22886310	25/25	8.35 ± 9.5	8.12 ± 7.2	7
Balci M. M. 2012	Turkey	22918198	61/33	63 ± 25*	16 ± 7	7
Feng X. 2012	China	22339491	132/108	3.84 ± 1.25	2.76 ± 0.91	8
Aihara K. 2011	Japan	21402472	150/20	2.1 ± 3.2*	1.4 ± 2.7	7
Guasti L. 2011	Italy	19924457	16/11	2.98 ± 2.7	4.81 ± 4.72	6
Kaditis A.G. 2010	Greece	20575100	84/22	2.2 ± 2.9*	1.3 ± 1.6	7
Bhushan B. 2009	India	18805681	62/46	3.6 ± 2	1.4 ± 1.4	8
Cofta S. 2009	Poland	20156725	40/14	2.5 ± 1.34*	2 ± 1.02	7
Kapsimalis F. 2008	Greece	18365276	52/15	3.5 ± 3*	1.9 ± 1.0	8
Takahashi K.I. 2008	Japan	18199002	41/12	1.72 ± 1.47	0.87 ± 0.96	7
Chung S. 2007	Korea	17702269	68/22	1.2 ± 1.46*	0.63 ± 0.83	7
Saletu M. 2006	Austria	16511651	103/44	5.8 ± 6.5*	2.8 ± 4.6	6
Guilleminault C. 2004	US	15683141	146/54	4.64 ± 6.74	4.10 ± 2.1	6

P/C NO.: number of OSA patients (P) and controls (C) in each group. CRP (mg/L) level was expressed as mean ± standard deviation (SD). NOS represents Newcastle-Ottawa Scale which was used for article quality evaluation.

*: the presented CRP protein level in OSA patients for this paper was from severe group because the paper provides protein data of different severity.

Detailed information for each included article, please refer to Supplement Table 3.

Detailed bibliography for each included publication, please refer to Supplement Table 4.

#### CPAP can significantly reduce TNF-α level, but slightly affect CRP in OSA

Next, we compared the changes in TNF-α of four studies and CRP of eight studies in OSA participants between the CPAP group and the non-CPAP group (Table 3, Supplemental Table 5). First, we analysed there were no statistical differences at baselines between the two groups for both TNF-α (WMD [95%CI] = 4.21[−2.29, 10.70] pg/ml, *p* = .20) and CRP (WMD [95%CI] = 0.07[−0.19, 0.32] mg/l, *p* = .61). After CPAP intervention, compared with non-CPAP group, TNF-α was decreased significantly (WMD [95%CI]= −4.44 [−4.81, −4.07] pg/ml, *p* < .00001), while CRP was decreased slightly (WMD [95%CI]= −0.91 [−1.65, −0.17] mg/l, *p* = .02) (Table 4, Supplemental Figures 6–7). Sensitivity analysis suggested that the trend of reduction in CRP and TNF-α remain unchanged. The funnel plot results indicated no apparent publication bias (Supplemental Figures 8–9).

**Table 3.** Characteristics of TNF-α and CRP levels in OSA participants with interventions of CPAP and non-CPAP.

Study ID	Region	NO.	Group	CPAP		non-CPAP		Period (M)	NOS
				Baseline	Post	Baseline	Post		
Wang X 2020	China	54/54	AHI	42.86 ± 15.08	3.96 ± 0.76	43.14 ± 15.05	41.23 ± 13.32	3	8
			TNF-α	17.12 ± 0.99	12.66 ± 0.86	17.12 ± 1.04	17.09 ± 1.09		
Wang Y. 2015(1)	China	25/15	AHI	36.9 ± 11.6	3.9 ± 1.1	31.7 ± 10.9	31.4 ± 9.6	0.5	6
			TNF-α	71.6 ± 25.8	48.2 ± 14	59.1 ± 22.8	60.2 ± 20.5		
Arias M.A. 2008	Spain	13/12	AHI	/	/	/	/	3	9
			TNF-α	18.2 ± 12.6	15.7 ± 10.9	18.2 ± 12.6	17.9 ± 11.4		
Li Y 2008	China	33/28	AHI	45.7 ± 24.9	24.7 ± 12.7	31.4 ± 28.6	32.5 ± 28.1	2	6
			TNF-α	124.5 ± 13.4	103.9 ± 11.7	113.8 ± 18.9	112.1 ± 18.9		
Campos-Rodriguez F. 2019	Spain	120/127	AHI	35.9 (24.2-50.1)^a^	/	31.4 (20.3-47.2)^a^	/	3	8
			CRP	3.86 ± 2.18	3.58 ± 2.09	3.87 ± 2.12	3.69 ± 1.99		
Huang Z. 2016	China	37/33	AHI	28.5 ± 12.1	2.7 ± 1.5	29.4 ± 12.9	28.6 ± 12.0	12	8
			CRP	2.71 ± 1.8	1.69 ± 1.4	2.49 ± 2.40	2.39 ± 2.60		
Wu S.Q. 2016	China	68/68	AHI	/	/	/	/	6	8
			CRP	2.9 ± 1.68	1.04 ± 0.94	2.78 ± 1.91	2.92 ± 2.29		
Wang Y. 2015(2)	China	25/15	AHI	36.9 ± 11.6	3.9 ± 1.1	31.7 ± 10.9	31.4 ± 9.6	0.5	6
			CRP	8.4 ± 0.8	6 ± 1.3	8.4 ± 0.5	8.1 ± 0.7		
Ishida K. 2009	Japan	40/15	AHI	47.8 ± 4.3^b^	/	46.3 ± 6.4^b^	/	6.1 ± 0.5	7
			CRP	2.3 ± 2.53	1.6 ± 1.9	2.4 ± 1.94	2 ± 1.94		
Takahashi K.I. 2008	Japan	27/14	AHI	48.2 ± 14.8	/	49.1 ± 24.1	/	1	6
			CRP	1.78 ± 1.56	1.20 ± 1.17	1.61 ± 1.3	1.19 ± 0.91		
Drager L.F. 2007	Brazil	12/12	AHI	56 ± 22	4.5 ± 1.9	66 ± 22	/	4	8
			CRP	3.7 ± 1.8	2 ± 1.2	3.1 ± 2.8	3.3 ± 2.7		
Steiropoulos P. 2007	Greece	20/14	AHI	56.71 ± 27.55	3.14 ± 5.64	47.49 ± 26.42	45.09 ± 23.44	6	8
			CRP	7.2 ± 5.4	5.5 ± 4.7	4.8 ± 4.7	6.1 ± 5.0		

NO.: The number of CPAP OSA patients and non-CPAP controls; AHI: unit events/h and format expressed as means ± SD except a expressed as median (IQR), b expressed as mean ± SE; TNF-α: unit pg/mL and format expressed as means ± SD; CRP: unit mg/L and format expressed as means ± SD; Period is measured in months (M).

Detailed bibliography for each included publication, please refer to Supplement Table 5.

**Table 4.** Comparison of TNF-α and CRP levels with different interventions in OSA subjects.

Protein	NO. of articles (OSAs in CPAP/non-CPAP)	Comparison stage between two groups	WMD [95% CI]	*P* value
TNF-α	4 (125/109)	Baseline	4.21 [−2.29, 10.70]	.2
	4 (125/109)	Post treatment	−**4.44 [**−**4.81, −4.07]**	**<.00001**
CRP	8 (349/298)	Baseline	0.07 [−0.19, 0.32]	.61
	8 (349/298)	Post treatment	−**0.91 [**−**1.65, −0.17]**	**.02**

NO. of articles (OSAs in CPAP/non-CPAP): The number of included articles and the number of OSA patients in CPAP group and non-CPAP group. WMD [95% CI]: Weighted mean difference (WMD) and 95% confidence interval (95%CI).

### Causal associations between TNF-α and CRP levels and OSA

**Figure 3.**
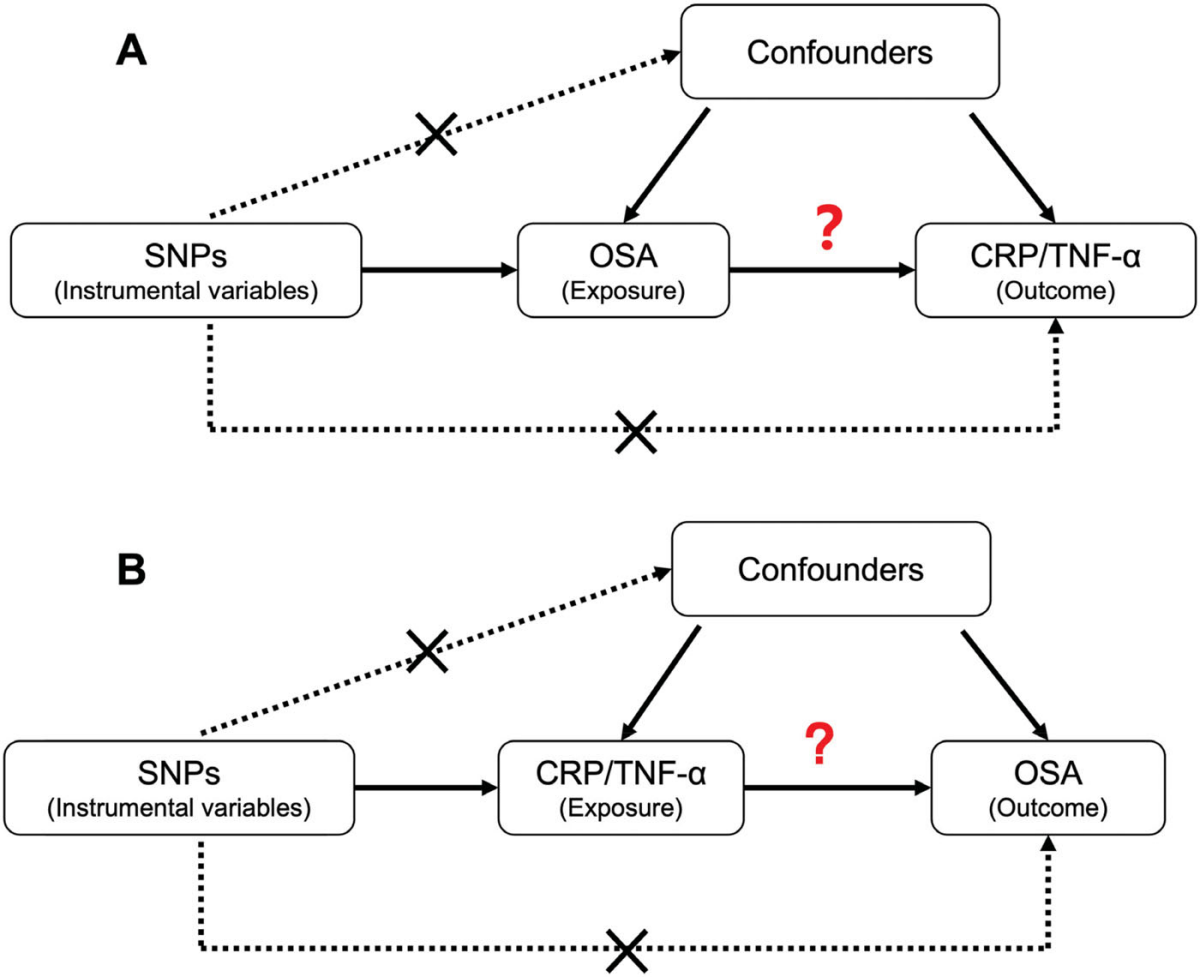
The design of Mendelian Randomization (MR) analysis to assess causality between OSA and inflammation proteins. (A) SNPs independently associated with OSA from GWAS summary statistic were used as instrumental variables to explore the causal effect of OSA on CRP or TNF-α. (B) SNPs independently associated with CRP or TNF-α from GWAS summary statistic were used as instrumental variables to explore the causal effect of CRP or TNF-α on OSA respectively. In addition to the association assumption, another two assumptions of MR include (1) SNPs are not associated with confounders between exposure and outcome, and (2) the effect from SNPs to outcome was exclusively through exposure.

Before we evaluated the causal associations between protein levels of TNF-α and CRP and OSA in two directions (Figure 3), we had confirmed the used instrumental variables were all strong with the F-statistic of 39.2 for OSA, 23.5 for TNF-α and 91.0 for CRP.

#### Causal analysis from OSA on elevated CRP and TNF-α

**Figure 4.**
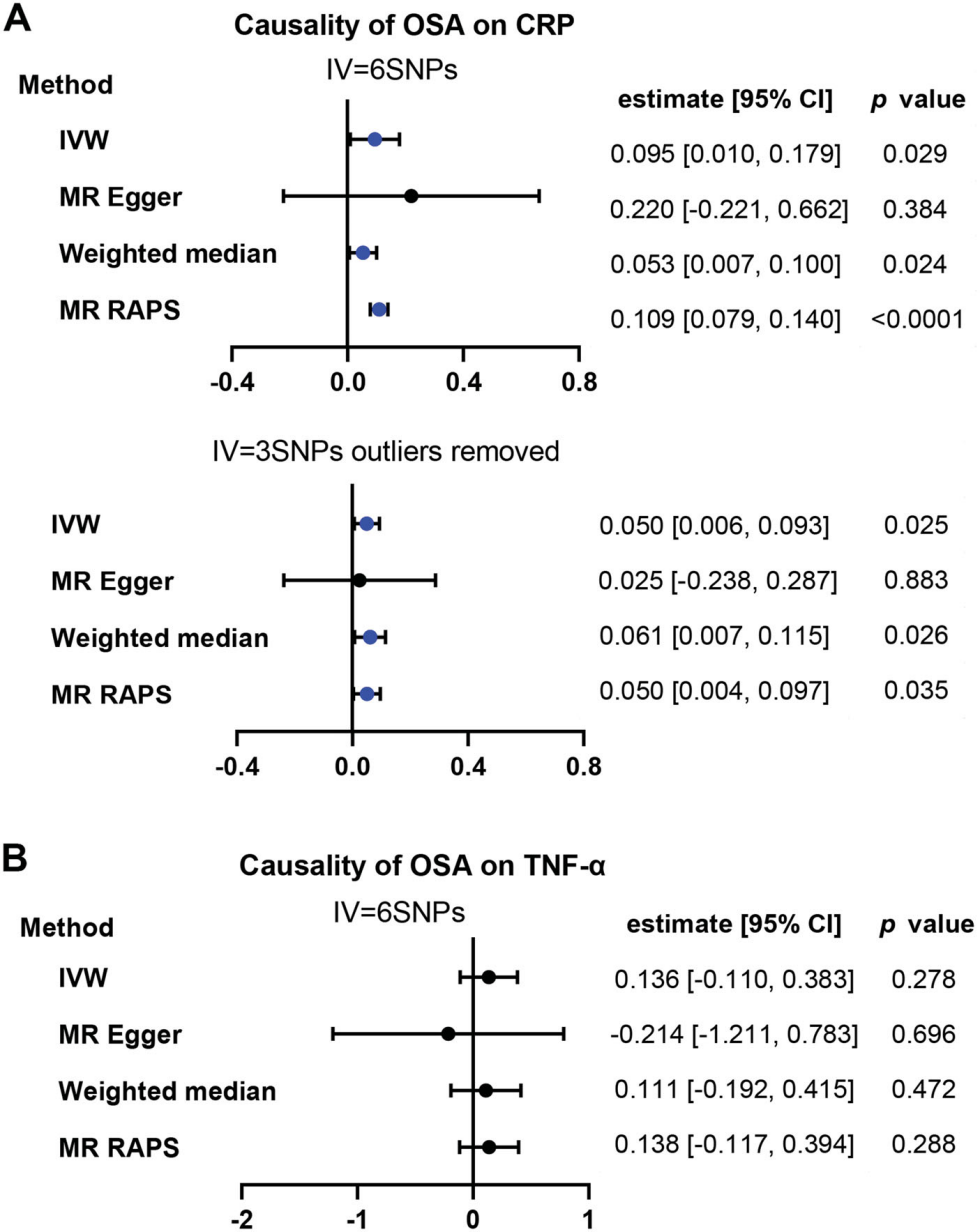
Causality analysis for OSA on CRP or TNF-α by Mendelian Randomisation (MR). Several MR methods were used to analyse the causality for OSA as exposure, CRP (A) and TNF-α (B) as outcomes respectively. If heterogeneity existed, outliers were removed and following similar analysis again. IV: instrumental variable. SNP: single nucleotide polymorphism. IVW: inverse variance weighted. MR RAPS: MR-Robust Adjusted Profile Score (RAPS). The statistically different results with *p*<.05 were shown in blue point otherwise were shown in black point if *p* >.05.

The primary method of IVW model showed that OSA was weakly associated with an increased risk of CRP (estimate: 0.095; 95% CI, [0.010, 0.179]; *p* = .029). Similar results were observed using Weighted median (estimate: 0.053; 95% CI, [0.007, 0.100]; *p* = .024) and MR RAPS (estimate: 0.109; 95% CI, [0.079, 0.140]; *p* = 1.98 × 10^−12^) except for MR-Egger (estimate: 0.220; 95% CI, [−0.221, 0.662]; *p* = .384). There was no evidence of pleiotropy (MR-Egger intercept −0.0112, standard error (SE) 0.0196, *p* = .599). However, there was high heterogeneity (*Q* = 46.16, *p* = 8.43 × 10^−09^). After removal the identified outliers of SNPs, there was no heterogeneity (*Q* = 0.548, *p* = .761) and the results were still significant with similar trends as before (Figure 4(A)). However, as for TNF-α, we did not observe any significant causal effect of OSA on TNF-α (Figure 4(B)).

#### Reverse analysis: Causal effect of CRP and TNF-α on OSA

**Figure 5.**
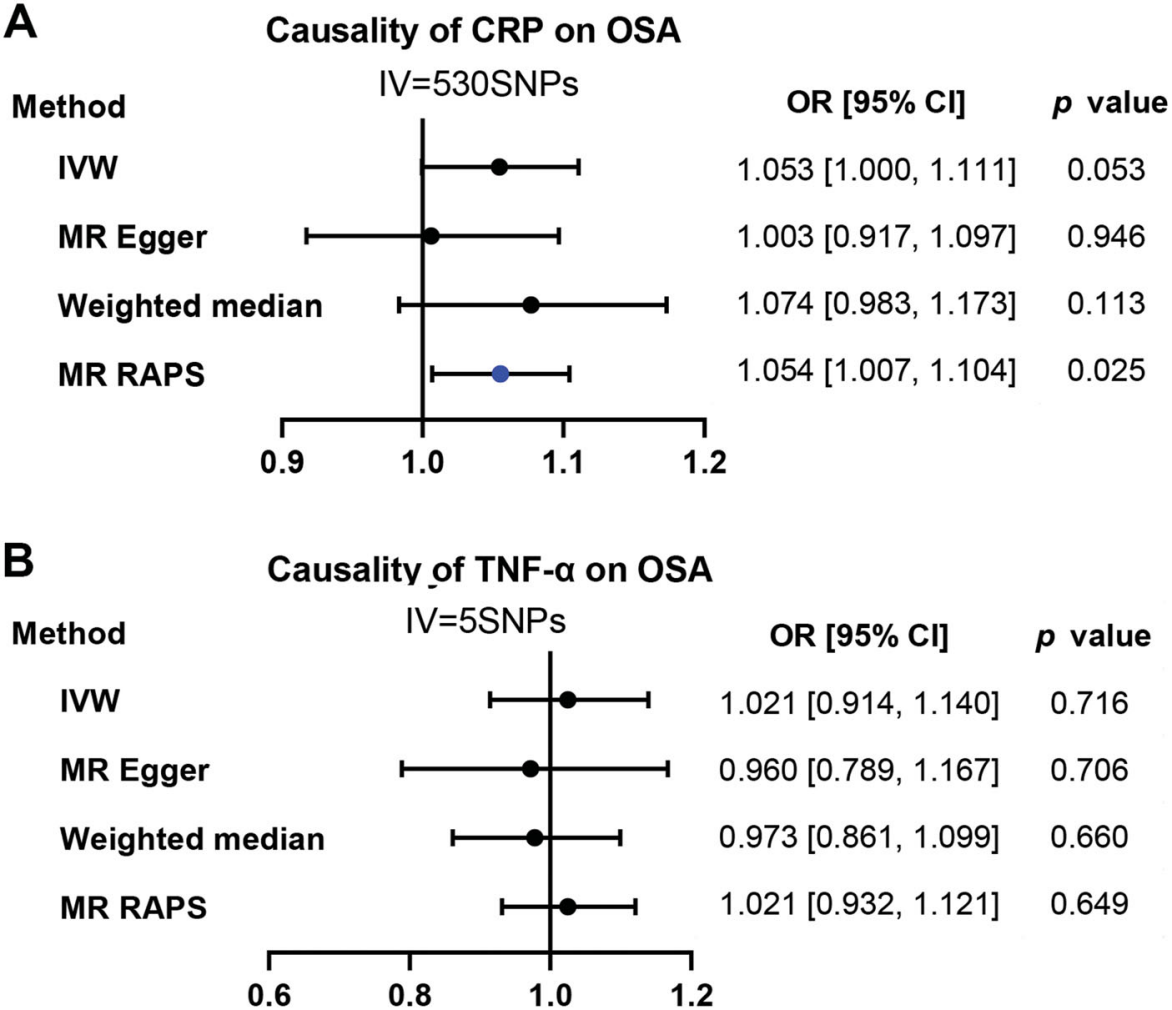
Causality analysis for CRP and TNF-α on OSA by Mendelian Randomization (MR). Several MR methods were applied to test the causality for CRP (A) and TNF-α (B) as exposure individually, OSA as outcome. IV: instrumental variable. SNP: single nucleotide polymorphism. IVW: inverse variance weighted. MR RAPS: MR-Robust Adjusted Profile Score (RAPS). The statistically different results with *p*<.05 were shown in blue point otherwise were shown in black point if *p* >.05.

When using CRP as exposure and OSA as outcome, the *p* value of the primary method of IVW is almost significant (OR:1.053; 95% CI, [1.000, 1.111]; *p* = .053). MR RAPS instead of MR-Egger and Weighted median was positive (OR:1.054; 95% CI, [1.007, 1.104]; *p* = .025) (Figure 5(A)). As for TNF-α. We didn’t observe any positive results for all applied methods (Figure 5(B)).

## Discussion

Cumulative evidence supports that systematic inflammation may play a critical role in OSA. However, the relationship between the changes in inflammatory proteins and OSA is not completely clear. Through comparisons of protein differences and bidirectional causality analysis, the main findings of this study were that patients with OSA tended to have increased CRP and TNF-α levels, and elevated CRP and TNF-α were positively associated with OSA severity. Besides, effective CPAP intervention can reduce abnormal TNF-α in OSA, but this effect on CRP was mild. Moreover, OSA was found to be a suggestive causality for elevated CRP by MR analysis. And the reverse causal effect of CRP on OSA may not be excluded. But there was no causality between OSA and TNF-α.

As expected, the levels of CRP and TNF-α were higher in OSA and positively associated with OSA severity. Both cellular experiments and histological biopsy suggested that OSA led to vascular inflammation with increasing CRP and TNF-α [31,32]. Consistent with our findings, a meta-analysis including 15 studies concluded that the level of CRP was higher in patients with severe OSA than in control subjects [14]. In addition to AHI, blood oxygen desaturation in OSA was also reported associated with CRP levels [33,34]. These findings support a strong link between inflammation and OSA. However, many confounding factors confuse our understanding of the association between inflammation and OSA. For instance, obesity is the most common risk factor for OSA [35] and obesity has also been shown to have an impact on cytokines including CRP and TNF-α [36]. It may confuse people whether high levels of inflammation proteins are the results of obesity or of OSA [37–39].

In this study, we used MR to overcome the influence of unmeasured confounders from observational study, and we found the suggestive causality from OSA on CRP through several complementary MR methods. There are several pathophysiologic mechanisms implying such causal relationship. Recurrent closure and narrowing of the upper airway can result in inflammation of mucosa [40,41]. And IH in OSA can lead to reactive oxygen species which activates inflammation pathways such as NF-kB^5^. Recent research reported that OSA caused significant alteration in serum extracellular microvesicles protein composition including CRP which may participate in OSA-related injury [42]. Interestingly, our result of mild reduction of CRP after CPAP intervention seemed to indirectly confirm the causality from OSA to CRP (Table 4). To be noticed, we can only say that CPAP can slightly reduce inflammation levels, but cannot prove whether it can be reduced to normal levels. A possible explanation for the mild reduction in CRP after CPAP therapy may be the existence of comorbidities such as obesity, arterial hypertension and diabetes in our included patients [43–45], whereas an RCT which only collected OSA free of any comorbidities reported a significant reduction in CRP [46]. These results suggested that the contributors to abnormal inflammation in OSA maybe not only OSA itself but also some comorbidities, and the latter may even mask the decrease in CRP after CPAP treatment [44,47].

In our reverse direction about causality from CRP to OSA, the almost significant association (OR:1.053; 95% CI, [1.000, 1.111]; *p* = .053) suggested we cannot exclude the possibility that CRP may lead to OSA. Consistently, a current longitudinal study reported that CRP was prospectively associated with increased OSA risk [48]. Besides that Gaines et al. reported that CRP is the mediation between obesity and OSA, indicating that the release of CRP by visceral adipocytes plays a causative role in the development of OSA [49]. According to the findings from bidirectional MR, there may exist a mild vicious cycle between OSA and CRP which needed further research to confirm.

The strengths of our study were that we explore the associations between OSA and CRP and TNF-α levels from multiple aspects including protein levels related to OSA-severity and CPAP treatment, and causal associations between them. Besides, using random distribution of genetic variation, the applied MR method can overcome potential confounding and reverse causation that may bias estimates from observational studies [21]. In the absence of large-scale and long-term RCT data, our findings provide useful data to estimate the causality between OSA and inflammation.

Despite the enlightening findings, this comprehensive analysis does leave several limitations. First, some related publications were not included in the meta-analysis because their data formats, like median and quartile, were not compatible with other included papers (mean and SD). Second, the diagnosis of OSA GWAS was from medical electronic records, which may have selection bias and impaired MR power. Because only people with obvious clinical symptoms will go to the hospital, and those with subclinical symptoms may be mistakenly included in the control group. Third, the sample size of TNF-α GWAS was relatively small which may be a reason why we did not find a causal relationship between TNF-α and OSA.

## Conclusions

In conclusion, our results confirmed that both CRP and TNF-α were increased in OSA and these increasing trends were severity-dependent. Though gently, elevated CRP and TNF-α can be reduced by effective CPAP treatment. In addition, MR analysis revealed suggestive causal direction from OSA to elevated CRP but not TNF-α. Further researches are needed to verify these findings.

## Supplementary Material

Supplemental Material

## Data Availability

Data is available on request to the corresponding author.
