# Supplementary material for: The causal relationships between obstructive sleep apnea and elevated CRP and TNF-α protein levels
Source: Ann Med. 2022 Jun 2;54(1):1578–89. doi: 10.1080/07853890.2022.2081873 (PMC9176672; doi:10.1080/07853890.2022.2081873)
Supplement: Supplemental Material [file IANN_A_2081873_SM7792.zip › Supplemental files/20220512_Supplementary_Methods_cleanV.pdf]

Supplementary material 1:

Supplemental Methods

Meta-analysis of association between CRP, TNF-α and OSA

A comprehensive analysis was conducted following guidelines Preferred Reporting Items for Systematic Reviews and Meta-Analyses (PRISMA)[1].

1.1 Search Strategy

This comprehensive search was performed using PubMed, Embase, Web of Science, and Cochrane Library by two authors independently. The searched terms were ((sleep apnea) OR OSA OR OSAS OR OSAHS) and (TNF OR (tumor necrosis factor)), ((sleep apnea) OR OSA OR OSAS OR OSAHS) and (CRP OR (C reactive protein)) up to December 04, 2021. The references of the enrolled studies were manually evaluated by two researchers independently and disagreements were resolved through discussion.

1.2 Inclusion criteria and exclusion criteria

We defined the inclusion criteria according to PICOS (participants, interventions, controls, outcomes and studies) as follows:

For different proteins expression, P: All participants were needed to diagnose OSA by questionnaire or polysomnography with an apnea hypopnea index (AHI) > 5 events/h in adults, and AHI > 1 events/h in children, or other accepted criteria without age, sex, BMI, and detection method restrictions. I: ELISA, multiplex assays, quantitative sandwich enzyme immunoassay kits, and other methods to detect the protein levels. C: non-OSA defined as AHI < 5 events/h in adults and AHI < 1 events/h in children, or other accepted criteria. O: Studies must report sufficient data about TNF-α and CRP levels in the format as mean ± standard deviation (SD). The original measurement unit of CRP must express as mg/L or mg/dl and that of TNF-α expressed as pg/ml. S: case-control study for different protein levels,

For CPAP treatment, P: same as described above. I: For CPAP, the period of follow-up of CPAP was at least 2 weeks. C: The non-CPAP was defined as OSA patients who didn't be treated with CPAP or received conservative therapy(dietary and sleep

hygiene counseling) or sham CPAP(≤pressure of 4 cm H2O) or poor compliance to CPAP. O: the study including both CPAP/non-CPAP proteins values for CPAP treatment in the format as mean ± standard deviation (SD) or standard error (SE). S: clinical trials

And the exclusion criteria were: (1) duplicated publications; (2) no original research (reviews, letters, editorials and conference abstract); (3) non-OSA disease; (4) genotyping studies.

1.3 Data extraction and quality Assessments

The data were extracted by two investigators. Summary statistics involved the basic study information and characteristics of participants were recorded in supplementary. Basic information involved the first author's name, year of publication, region, case type, age, body mass index (BMI), gender distribution and AHI of both groups, detection method, sample sources, and data presentation format mean ± SD.

Finally, the Newcastle-Ottawa Scale (NOS) assessed the quality of the studies included in this analysis[2].

1.4 Data analysis

All data were analyzed in the Review manager 5.3 (The Nordic Cochrane Centre, The Cochrane Collaboration, London, UK). First part, we explored whether the protein level of TNF-α and CRP are associated with OSA severity. Studies were categorized into 3 subgroups according to AHI: mild OSA (AHI 5 to <15), moderate OSA (AHI 15 to <30) and severe OSA (AHI ≥30) [3]. If a study contained multiple groups of severity, we selected the most severe one into the analysis. Finally, subgroup analyses were conducted on the studies evaluating TNF-α, CRP.

Second part, to analyze whether TNF-α and CRP levels could be reduced after CPAP therapy in OSA, we compared the efficacy of CPAP and non-CPAP simultaneously (baseline stage of CPAP VS non-CPAP; post treatment stage of CPAP VS non-CPAP) to observe level changes in different proteins over time in different populations.

The measurement unit of CRP and TNF-α were standardized. For CRP, mg/dl was converted to mg/l, TNF-α was pg/ml. As for continuous outcomes, the weighted mean differences (WMD) and 95% confidence interval (CI) were used as measures of effect

between the two groups in this study. We calculate an I^2 statistic to estimate heterogeneity. If I2 > 50%, the data were pooled by random effect model, otherwise by fixed effect model. We also performed a sensitivity analysis by removing article one by one to see its effect on the p value. Moreover, a funnel diagram was conducted to evaluate publication bias.

Reference

1. Moher D, Liberati A, Tetzlaff J, Altman DG. Preferred reporting items for systematic reviews and meta-analyses: the PRISMA statement. *J Clin Epidemiol.* 2009;62:1006-1012.
2. Patel SR, Larkin EK, Redline S. Shared genetic basis for obstructive sleep apnea and adiposity measures. *Int J Obes (Lond).* 2008;32:795-800.
3. Ho V, Crainiceanu CM, Punjabi NM, Redline S, Gottlieb DJ. Calibration Model for Apnea-Hypopnea Indices: Impact of Alternative Criteria for Hypopneas. *Sleep.* 2015;38:1887-1892.
