# Supplementary material for: The causal relationships between obstructive sleep apnea and elevated CRP and TNF-α protein levels
Source: Ann Med. 2022 Jun 2;54(1):1578–89. doi: 10.1080/07853890.2022.2081873 (PMC9176672; doi:10.1080/07853890.2022.2081873)
Supplement: Supplemental Material [file IANN_A_2081873_SM7792.zip › Supplemental files/20220528_Supplementary Figures[AU] (1).pdf]

Supplemental material 3:

Supplemental figures.

Study or Subgroup	Experimental Mean	Experimental SD	Experimental Total	Control Mean	Control SD	Control Total	Weight	Mean Difference IV, Random, 95% CI
Nobili V. 2014	2.2	6.6	39	6.8	2	26	3.7%	-4.60 [-6.81, -2.39]
Smith D.F. 2017	6	4.1	42	10.1	45.4	90	1.0%	-4.10 [-13.56, 5.36]
Chuang H.H. 2020	44.9	19.3	11	45.9	19.2	24	0.5%	-1.00 [-14.75, 12.75]
Rogers V.E. 2018	4.5	3.18	20	5.24	3.25	7	3.4%	-0.74 [-3.52, 2.04]
Alexopoulos E.I. 2013	0.63	0.2	24	0.63	0.17	22	4.4%	0.00 [-0.11, 0.11]
Unuvar Dogan F. 2014	0.128	0.15	33	0.108	0.027	24	4.4%	0.02 [-0.03, 0.07]
Zhang Z. 2017	3.01	0.21	50	2.89	0.23	52	4.4%	0.12 [0.03, 0.21]
Gamsiz-Isik H. 2017	11.5	3.11	83	11.25	4	80	4.2%	0.25 [-0.85, 1.35]
Sahlman J. 2010	1.54	1.75	84	1.17	1.58	40	4.3%	0.37 [-0.25, 0.99]
Kobayashi K. 2006	1.11	0.46	35	0.62	0.44	16	4.4%	0.49 [0.23, 0.75]
Tomiyama H. 2008	2.2	0.6	28	1.7	0.5	15	4.4%	0.50 [0.16, 0.84]
Antonopoulou S. 2008	1.4	0.9	45	0.64	0.3	25	4.4%	0.76 [0.47, 1.05]
Kim J. 2010	15.32	6.8	28	14.4	4.13	22	3.2%	0.92 [-2.13, 3.97]
Minoguchi K. 2004	2.34	0.54	12	1.12	0.39	12	4.4%	1.22 [0.84, 1.60]
Ciftci T. U. 2004	4.6	3.39	43	3.29	2.13	22	4.1%	1.31 [-0.04, 2.66]
Medeiros C.A.M. 2012	2.09	7.3	35	0.32	0.77	15	3.6%	1.77 [-0.68, 4.22]
Steiropoulos P. 2010	6.72	3.72	38	3.94	1.34	23	4.1%	2.78 [1.48, 4.08]
Akinnusi M. 2013	6.83	2.55	25	1.57	0.44	18	4.2%	5.26 [4.24, 6.28]
Bilal N. 2021	16.11	2.2	10	10.58	1.71	30	4.0%	5.53 [4.04, 7.02]
Chen V.G. 2020	50.39	25.86	17	44.41	13.77	17	0.5%	5.98 [-7.95, 19.91]
Bozic J. 2018	8.67	2.41	25	2.35	1.25	25	4.2%	6.32 [5.26, 7.38]
Yang D. 2013	12.55	8.09	25	5.12	1.23	25	3.1%	7.43 [4.22, 10.64]
Ciccone M.M. 2014	20.09	5.39	80	12.53	3.48	40	4.0%	7.56 [5.96, 9.16]
Jin F. 2017	37.67	0.21	100	29.15	1.74	50	4.3%	8.52 [8.04, 9.00]
Ming H. 2019	31.2	5.3	684	12.1	1.1	192	4.4%	19.10 [18.67, 19.53]
Alberti A. 2003	26.9	3.45	18	6.5	1.55	20	3.9%	20.40 [18.67, 22.13]
Leon-Cabrera S. 2015	337.9	67.8	29	306.9	38.9	13	0.1%	31.00 [-1.50, 63.50]
Chen J. 2007	127.6	37.76	100	93.13	32.14	40	0.6%	34.47 [22.06, 46.88]
De Santis S. 2015	122.2	12	26	80.2	18.3	24	1.1%	42.00 [33.35, 50.65]
Li Y 2009	132.1	10.8	24	87.3	6.1	22	2.2%	44.80 [39.78, 49.82]
Constantinidis J. 2008	124.64	96.7	13	78.8	50.1	12	0.0%	45.84 [-13.88, 105.56]
Kanbay A. 2008	114.15	144.15	106	34.25	13.1	32	0.1%	79.90 [52.09, 107.71]
Ji L. 2020	138.9	105.3	27	34.9	21.9	21	0.1%	104.00 [63.19, 144.81]
Liu H. 2000	299.09	43.57	22	101.88	21.27	16	0.2%	197.21 [176.23, 218.19]
**Total (95% CI)**			**1981**			**1112**	**100.0%**	**5.86 [4.80, 6.93]**

Heterogeneity: Tau² = 6.75; Chi² = 10493.18, df = 33 (P < 0.00001); I² = 100%
Test for overall effect: Z = 10.78 (P < 0.00001)

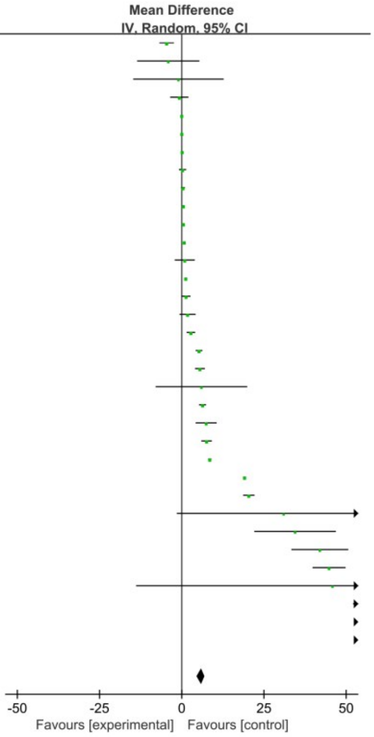

Supplemental figure 1. Forest plot of TNF-α concentration difference between OSA patients and controls.

Study or Subgroup	Experimental Mean	Experimental SD	Experimental Total	Control Mean	Control SD	Control Total	Weight	Mean Difference IV, Random, 95% CI
Guasti L. 2011	2.98	2.7	16	4.81	4.72	11	1.2%	-1.83 [-4.92, 1.26]
Bezerra P.C 2013	6.7	5	27	8	7.9	21	1.0%	-1.30 [-5.17, 2.57]
Azar C. 2020	9.3	6.5	99	9.3	9.3	55	1.3%	0.00 [-2.77, 2.77]
Zhang D. 2017	2.19	1.94	38	2.09	3.46	10	1.5%	0.10 [-2.13, 2.33]
Van Eyck A. 2014	1.95	3.63	15	1.8	3.6	85	1.6%	0.15 [-1.84, 2.14]
Horvath P. 2018	4.2	3.7	50	4	1.8	26	1.9%	0.20 [-1.04, 1.44]
Jung J.H. 2021	0.6	0.74	27	0.38	0.33	21	2.1%	0.22 [-0.09, 0.53]
Nural S. 2013	8.35	9.5	25	8.12	7.2	25	0.8%	0.23 [-4.44, 4.90]
Wang Y. 2015	8.4	0.7	47	8.1	0.8	28	2.1%	0.30 [-0.06, 0.66]
Chen D.D. 2020	1.22	0.34	37	0.81	0.22	17	2.1%	0.41 [0.26, 0.56]
Nakabayashi K. 2017	0.96	2.49	134	0.52	1.41	109	2.1%	0.44 [-0.06, 0.94]
Xu Q. 2017	1.47	1.6	33	0.97	1.22	30	2.1%	0.50 [-0.20, 1.20]
Cofta S. 2009	2.5	1.34	13	2	1.02	14	2.0%	0.50 [-0.40, 1.40]
Mônico-Neto M. 2018	3.4	0.4	123	2.9	0.5	211	2.1%	0.50 [0.40, 0.60]
Guilleminault C. 2004	4.64	6.74	146	4.1	2.1	54	1.9%	0.54 [-0.69, 1.77]
Chung S. 2007	1.2	1.46	40	0.63	0.83	22	2.1%	0.57 [-0.00, 1.14]
Aihara K. 2011	2.1	3.2	73	1.4	2.7	20	1.9%	0.70 [-0.69, 2.09]
Tanriverdi H. 2016	2.8	3.8	53	2.05	3.38	24	1.7%	0.75 [-0.95, 2.45]
Ciccone M.M. 2014	1.84	0.67	54	1.08	0.53	40	2.1%	0.76 [0.52, 1.00]
Takahashi K.I. 2008	1.72	1.47	41	0.87	0.96	12	2.1%	0.85 [0.14, 1.56]
Zhang D.M. 2018	2.09	1.8	30	1.19	1.14	20	2.0%	0.90 [0.08, 1.72]
Kaditis A.G. 2010	2.2	2.9	29	1.3	1.6	22	1.9%	0.90 [-0.35, 2.15]
Zhang H. 2016	4.25	0.61	41	3.32	0.35	19	2.1%	0.93 [0.69, 1.17]
Feng X. 2012	3.84	1.25	132	2.76	0.91	108	2.1%	1.08 [0.81, 1.35]
Masood R.K. 2017	1.72	1.47	90	0.63	0.35	63	2.1%	1.09 [0.77, 1.41]
Chien M.Y. 2020	2.3	1.4	20	1.2	0.6	20	2.1%	1.10 [0.43, 1.77]
Huang Y.S. 2016	1.9	0.44	47	0.41	0.48	32	2.1%	1.49 [1.28, 1.70]
Kapsimalis F. 2008	3.5	3	26	1.9	1	15	1.9%	1.60 [0.34, 2.86]
Chen Y. C. 2021	3.64	4.73	56	1.79	1.11	16	1.9%	1.85 [0.50, 3.20]
Voulgaris A. 2019	5.5	5.8	64	3.4	3.6	32	1.7%	2.10 [0.21, 3.99]
Brener A. 2020	6.4	2.3	14	4.3	2.2	44	1.9%	2.10 [0.73, 3.47]
Alonso-Álvarez M.L. 2017	4.71	10.95	62	2.54	2.6	51	1.3%	2.17 [-0.65, 4.99]
Bhatt S. P. 2021	3.7	2.12	190	1.5	0.71	57	2.1%	2.20 [1.85, 2.55]
Bhatt S.P. 2019	3.6	1.5	47	1.4	0.7	25	2.1%	2.20 [1.69, 2.71]
Uygur F. 2016	3.6	1.8	96	1.4	0.9	31	2.1%	2.20 [1.72, 2.68]
Bhushan B. 2009	3.6	2	62	1.4	1.4	46	2.1%	2.20 [1.56, 2.84]
Xie J.Y. 2020	3.14	2.77	59	0.82	1.21	34	2.0%	2.32 [1.50, 3.14]
Gamsiz-Isik H. 2017	4.95	8.95	83	2.38	1.8	80	1.6%	2.57 [0.60, 4.54]
Sanz-Rubio D. 2020	3.5	2.5	16	0.8	0.9	7	1.9%	2.70 [1.31, 4.09]
Zhang L. 2020	10.8	5.6	53	8	5.4	19	1.3%	2.80 [-0.06, 5.66]
Huang Y.S. 2020	3.37	6.04	55	0.42	0.23	32	1.8%	2.95 [1.35, 4.55]
Song T.J. 2017	9	28	117	6	15	94	0.6%	3.00 [-2.91, 8.91]
Israel L.P. 2013	4.5	2.1	25	1.5	1	24	2.0%	3.00 [2.08, 3.92]
Perticone M. 2021	6.5	6.4	65	3.5	2.4	100	1.8%	3.00 [1.37, 4.63]
Saletu M. 2006	5.8	6.5	51	2.8	4.6	44	1.5%	3.00 [0.76, 5.24]
Chu A.A. 2020	5.4	1.1	71	2	0.5	31	2.1%	3.40 [3.09, 3.71]
Kunos L. 2018	6.3	13	45	2.8	2.4	31	1.0%	3.50 [-0.39, 7.39]
Morell-Garcia D. 2020	6	16	38	2	2	76	0.7%	4.00 [-1.11, 9.11]
Li F. 2016	6.45	7.25	68	2.33	2.82	35	1.6%	4.12 [2.16, 6.08]
Rong W. 2021	7.02	0.34	42	2.88	0.22	42	2.1%	4.14 [4.02, 4.26]
Cao Z. 2016	14.2	3.3	53	9.2	1.2	56	2.0%	5.00 [4.06, 5.94]
Kim J. 2016	14.7	16	251	9.7	12.2	973	1.6%	5.00 [2.88, 7.12]
Fouda N. 2014	6.7	0.6	14	1.5	0.3	16	2.1%	5.20 [4.85, 5.55]
Bauça J.M. 2019	41.34	60.87	209	36.08	61.19	152	0.2%	5.26 [-7.50, 18.02]
Liu C.D. 2017	7.5	3.5	97	1.6	0.7	30	2.1%	5.90 [5.16, 6.64]
Cilekar S. 2021	31.2	31.5	20	21	12.1	40	0.1%	10.20 [-4.11, 24.51]
Pusuroglu H. 2017	52	59	87	36	41	21	0.1%	16.00 [-5.48, 37.48]
Cignarelli A. 2021	82	72	68	66	71	22	0.0%	16.00 [-18.25, 50.25]
Aydin S. 2018	42	38	47	20	21	17	0.1%	22.00 [7.25, 36.75]
Pelaia C. 2021	48	38	162	25	13	40	0.4%	23.00 [15.90, 30.10]
Wang L.J. 2021	52.81	27	122	23.99	10.91	130	0.7%	28.82 [23.67, 33.97]
Yardim-Akaydin S. 2014	62.3	54.7	139	33.2	4.9	27	0.3%	29.10 [19.82, 38.38]
Jin F. 2017	49.64	21.66	100	6.37	1.3	50	0.9%	43.27 [39.01, 47.53]
Balci M. M. 2012	63	25	31	16	7	33	0.3%	47.00 [37.88, 56.12]
**Total (95% CI)**			**4285**			**3692**	**100.0%**	**2.66 [2.15, 3.17]**

Heterogeneity: $Tau^2 = 3.20$; $Chi^2 = 4123.96$, df = 63 ($P < 0.00001$); $I^2 = 98\%$
Test for overall effect: $Z = 10.15$ ($P < 0.00001$)

Mean Difference
IV, Random, 95% CI

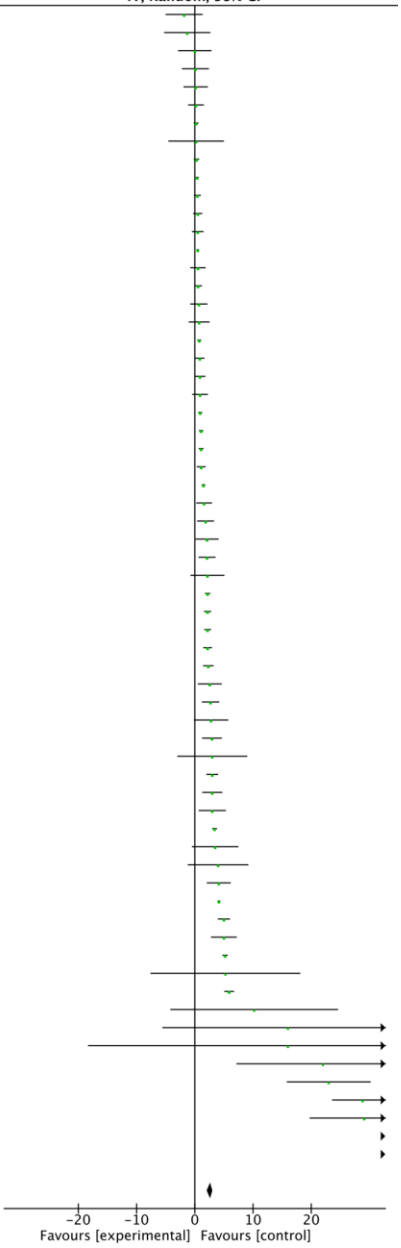

Supplemental figure 2. Forest plot of CRP concentration difference between OSA patients and controls.

Study or Subgroup	Experimental Mean	Experimental SD	Experimental Total	Control Mean	Control SD	Control Total	Weight	Mean Difference IV, Random, 95% CI
**2.1.1 mild OSA VS no OSA**								
Smith D.F. 2017	5.9	6	23	10.1	45.4	90	0.4%	−4.20 [−13.89, 5.49]
Tomiyama H. 2008	1.5	0.3	10	1.7	0.5	15	15.2%	−0.20 [−0.51, 0.11]
Alexopoulos E.I. 2013	0.65	0.18	22	0.63	0.17	22	15.8%	0.02 [−0.08, 0.12]
Nobili V. 2014	6.9	2.1	26	6.8	2	26	10.0%	0.10 [−1.01, 1.21]
Gamsiz-Isik H. 2017	11.5	4.6	16	11.25	4	80	4.2%	0.25 [−2.17, 2.67]
Sahlman J. 2010	1.54	1.75	84	1.17	1.58	40	13.5%	0.37 [−0.25, 0.99]
Medeiros C.A.M. 2012	0.84	1.8	15	0.32	0.77	15	10.9%	0.52 [−0.47, 1.51]
Minoguchi K. 2004	1.8	0.43	12	1.12	0.39	12	15.1%	0.68 [0.35, 1.01]
Bilal N. 2021	11.75	2.64	10	10.58	1.71	30	6.5%	1.17 [−0.58, 2.92]
Ciccone M.M. 2014	14.42	3.29	26	12.53	3.48	40	6.9%	1.89 [0.23, 3.55]
Li Y 2009	102.3	11.3	22	87.3	6.1	22	1.1%	15.00 [9.63, 20.37]
Ji L. 2021	52.5	50.9	20	34.9	21.9	21	0.1%	17.60 [−6.59, 41.79]
De Santis S. 2015	120	14.1	9	80.2	18.3	24	0.2%	39.80 [28.03, 51.57]
Constantinidis J. 2008	124.64	96.7	13	78.8	50.1	12	0.0%	45.84 [−13.88, 105.56]
**Subtotal (95% CI)**			**308**			**449**	**100.0%**	**0.67 [0.09, 1.25]**
Heterogeneity: $Tau^2 = 0.55$; $Chi^2 = 103.33$, df = 13 ($P < 0.00001$); $I^2 = 87\%$								
Test for overall effect: Z = 2.26 (P = 0.02)								
**2.1.2 moderate OSA VS no OSA**								
Smith D.F. 2017	6	4.1	42	10.1	45.4	90	0.5%	−4.10 [−13.56, 5.36]
Gamsiz-Isik H. 2017	10.3	2.59	67	11.25	4	80	9.4%	−0.95 [−2.02, 0.12]
Alexopoulos E.I. 2013	0.63	0.2	24	0.63	0.17	22	12.2%	0.00 [−0.11, 0.11]
Unuvar Dogan F. 2014	0.128	0.15	33	0.108	0.027	24	12.2%	0.02 [−0.03, 0.07]
Kim J. 2010	14.56	5.61	9	14.4	4.13	22	2.3%	0.16 [−3.89, 4.21]
Tomiyama H. 2008	1.9	0.5	12	1.7	0.5	15	11.8%	0.20 [−0.18, 0.58]
Minoguchi K. 2004	2.34	0.54	12	1.12	0.39	12	11.8%	1.22 [0.84, 1.60]
Bilal N. 2021	11.94	1.35	10	10.58	1.71	30	9.6%	1.36 [0.32, 2.40]
Bozic J. 2018	5.79	1.44	25	2.35	1.25	25	10.7%	3.44 [2.69, 4.19]
Akinnusi 2013	6.83	2.55	25	1.57	0.44	18	9.6%	5.26 [4.24, 6.28]
Ciccone M.M. 2014	22.83	3.85	54	12.53	3.48	40	7.8%	10.30 [8.81, 11.79]
Ji L. 2021	61.2	45.1	20	34.9	21.9	21	0.1%	26.30 [4.43, 48.17]
Li Y 2009	125	11.9	22	87.3	6.1	22	1.3%	37.70 [32.11, 43.29]
De Santis S. 2015	123.4	11.1	17	80.2	18.3	24	0.6%	43.20 [34.18, 52.22]
**Subtotal (95% CI)**			**372**			**445**	**100.0%**	**2.64 [1.95, 3.33]**
Heterogeneity: $Tau^2 = 1.00$; $Chi^2 = 677.80$, df = 13 ($P < 0.00001$); $I^2 = 98\%$								
Test for overall effect: Z = 7.54 (P < 0.00001)								
**2.1.3 servere OSA VS no OSA**								
Nobili V. 2014	6.9	2.3	13	6.8	2	26	14.6%	0.10 [−1.37, 1.57]
Tomiyama H. 2008	2.2	0.6	28	1.7	0.5	15	14.8%	0.50 [0.16, 0.84]
Kim J. 2010	15.32	6.8	28	14.4	4.13	22	13.8%	0.92 [−2.13, 3.97]
Medeiros C.A.M. 2012	2.09	7.3	35	0.32	0.77	15	14.2%	1.77 [−0.68, 4.22]
Bilal N. 2021	16.11	2.2	10	10.58	1.71	30	14.6%	5.53 [4.04, 7.02]
Bozic J. 2018	8.67	2.41	25	2.35	1.25	25	14.7%	6.32 [5.26, 7.38]
Li Y 2009	132.1	10.8	24	87.3	6.1	22	12.3%	44.80 [39.78, 49.82]
Ji L. 2021	138.9	105.3	27	34.9	21.9	21	1.0%	104.00 [63.19, 144.81]
**Subtotal (95% CI)**			**190**			**176**	**100.0%**	**8.76 [4.52, 12.99]**
Heterogeneity: $Tau^2 = 31.45$; $Chi^2 = 453.75$, df = 7 ($P < 0.00001$); $I^2 = 98\%$								
Test for overall effect: Z = 4.05 (P < 0.0001)								

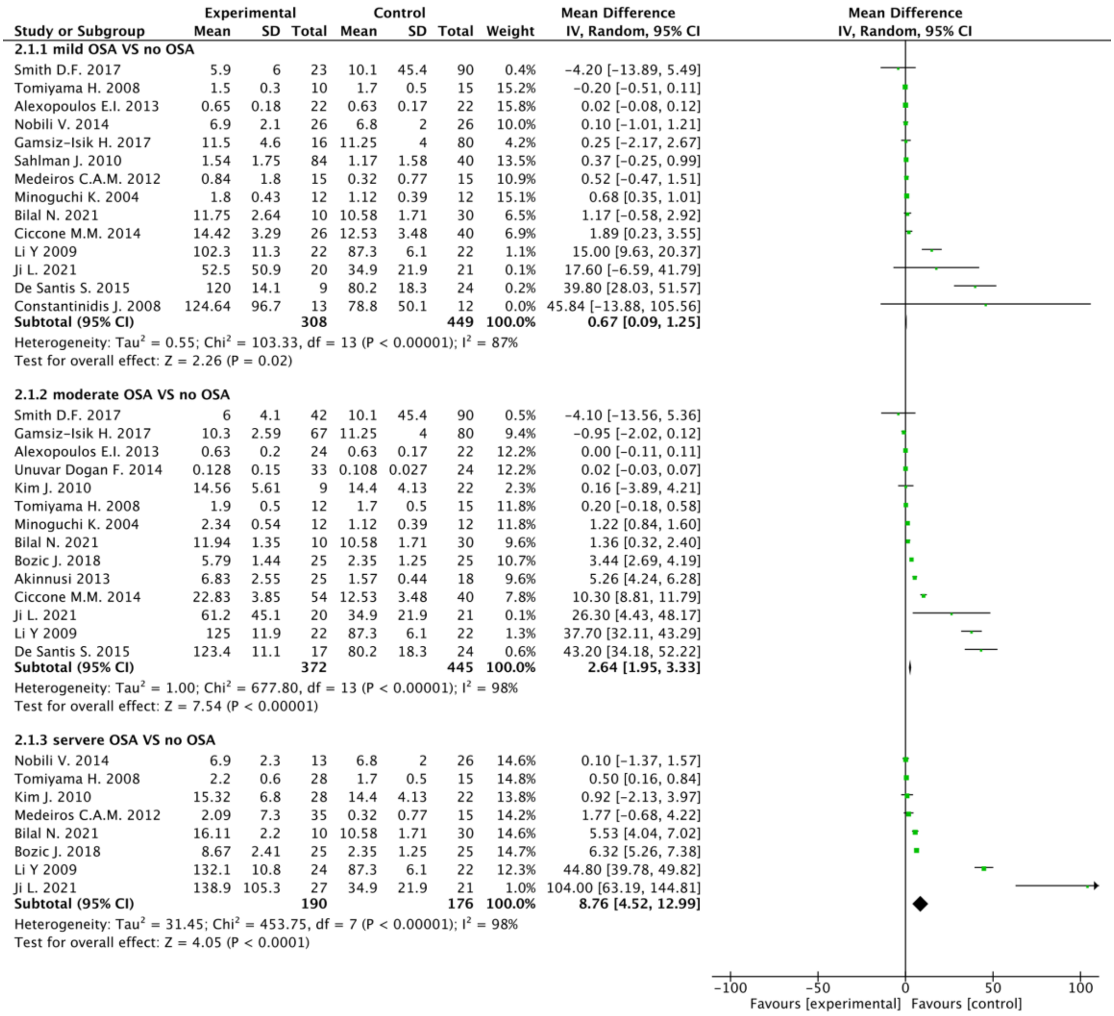

Supplemental figure 3. Forest plot of TNF-α concentration difference between mild, moderate, severe OSA patients and controls.

Study or Subgroup	Experimental Mean	Experimental SD	Experimental Total	Control Mean	Control SD	Control Total	Weight	Mean Difference IV, Random, 95% CI
**2.2.1 mild OSA VS no OSA**								
Song T.J. 2017	4	9	72	6	15	94	0.7%	−2.00 [−5.68, 1.68]
Cofta S. 2009	1.92	1.15	14	2	1.02	14	5.3%	−0.08 [−0.89, 0.73]
Jung J.H. 2021	0.34	0.33	29	0.38	0.33	21	7.9%	−0.04 [−0.23, 0.15]
Morell-Garcia D. 2020	2	2	61	2	2	76	5.9%	0.00 [−0.67, 0.67]
Ciccone M.M. 2014	1.32	0.48	26	1.08	0.53	40	7.7%	0.24 [−0.01, 0.49]
Van Eyck A. 2014	2.1	6.7	20	1.8	3.6	85	0.9%	0.30 [−2.73, 3.33]
Mônico-Neto M. 2018	3.2	0.4	201	2.9	0.5	211	8.0%	0.30 [0.21, 0.39]
Chung S. 2007	1.15	1.28	28	0.63	0.83	22	6.3%	0.52 [−0.07, 1.11]
Aihara K. 2011	2	4	25	1.4	2.7	20	1.9%	0.60 [−1.36, 2.56]
Masood R.K. 2017	1.31	0.85	60	0.63	0.35	63	7.7%	0.68 [0.45, 0.91]
Kapsimalis F. 2008	2.6	2	26	1.9	1	15	4.8%	0.70 [−0.22, 1.62]
Kaditis A.G. 2010	2.1	3.6	55	1.3	1.6	22	3.8%	0.80 [−0.36, 1.96]
Tanriverdi H. 2016	2.85	4.58	20	2.05	3.38	24	1.4%	0.80 [−1.62, 3.22]
Saletu M. 2006	3.6	4.1	27	2.8	4.6	44	1.8%	0.80 [−1.26, 2.86]
Uygur F. 2016	2.3	1.1	31	1.4	0.9	31	6.7%	0.90 [0.40, 1.40]
Pusuroglu H. 2017	37	31	34	36	41	21	0.0%	1.00 [−19.40, 21.40]
Sanz-Rubio D. 2020	2.6	2.8	19	1.3	1.2	31	3.3%	1.30 [−0.03, 2.63]
Bhushan B. 2009	2.7	2.2	27	1.4	1.4	46	4.8%	1.30 [0.38, 2.22]
Li F. 2016	3.68	6.56	28	2.33	2.82	35	1.2%	1.35 [−1.25, 3.95]
Zhang L. 2020	9.5	7.8	81	8	5.4	19	1.0%	1.50 [−1.46, 4.46]
Cao Z. 2016	10.8	2.4	74	9.2	1.2	56	6.1%	1.60 [0.97, 2.23]
Xie J.Y. 2020	2.44	3.97	48	0.82	1.21	34	3.7%	1.62 [0.43, 2.81]
Kim J. 2016	12	13.4	611	9.7	12.2	973	3.4%	2.30 [0.99, 3.61]
Gamsiz-Isik H. 2017	5.52	9.85	16	2.38	1.8	80	0.4%	3.14 [−1.70, 7.98]
Liu C.D. 2017	6.6	3.1	46	1.6	0.7	30	4.7%	5.00 [4.07, 5.93]
Pelaia C. 2021	30	22	93	25	13	40	0.3%	5.00 [−1.02, 11.02]
Balci M. M. 2012	22	24	30	16	7	33	0.1%	6.00 [−2.91, 14.91]
Cilekar S. 2021	33.6	32.7	50	21	12.1	40	0.1%	12.60 [2.79, 22.41]
Yardim-Akaydin S. 2014	60.6	63.7	36	33.2	4.9	27	0.0%	27.40 [6.51, 48.29]
**Subtotal (95% CI)**			**1888**			**2247**	**100.0%**	**0.93 [0.62, 1.25]**
Heterogeneity: $Tau^2 = 0.32$; $Chi^2 = 185.62$, df = 28 (P < 0.00001); $I^2 = 85\%$								
Test for overall effect: Z = 5.81 (P < 0.00001)								
**2.2.2 moderate OSA VS no OSA**								
Aihara K. 2011	1.3	2.2	52	1.4	2.7	20	4.0%	−0.10 [−1.43, 1.23]
Gamsiz-Isik H. 2017	2.5	1.5	67	2.38	1.8	80	7.2%	0.12 [−0.41, 0.65]
Jung J.H. 2021	0.51	0.65	31	0.38	0.33	21	8.2%	0.13 [−0.14, 0.40]
Van Eyck A. 2014	1.95	3.63	15	1.8	3.6	85	2.4%	0.15 [−1.84, 2.14]
Masood R.K. 2017	0.78	0.33	67	0.63	0.35	63	8.5%	0.15 [0.03, 0.27]
Chen D.D. 2020	1.04	0.26	36	0.81	0.22	17	8.5%	0.23 [0.10, 0.36]
Mônico-Neto M. 2018	3.4	0.4	123	2.9	0.5	211	8.5%	0.50 [0.40, 0.60]
Cofta S. 2009	2.52	1	13	2	1.02	14	6.2%	0.52 [−0.24, 1.28]
Ciccone M.M. 2014	1.84	0.67	54	1.08	0.53	40	8.2%	0.76 [0.52, 1.00]
Tanriverdi H. 2016	2.85	8.07	15	2.05	3.38	24	0.6%	0.80 [−3.50, 5.10]
Kaditis A.G. 2010	2.2	2.9	29	1.3	1.6	22	4.2%	0.90 [−0.35, 2.15]
Saletu M. 2006	4	7.9	25	2.8	4.6	44	1.0%	1.20 [−2.18, 4.58]
Uygur F. 2016	3.4	1.4	33	1.4	0.9	31	7.0%	2.00 [1.43, 2.57]
Bhushan B. 2009	4.1	0.8	9	1.4	1.4	46	6.6%	2.70 [2.04, 3.36]
Perticone M. 2021	6.5	6.4	65	3.5	2.4	100	3.1%	3.00 [1.37, 4.63]
Song T.J. 2017	9	28	117	6	15	94	0.4%	3.00 [−2.91, 8.91]
Li F. 2016	5.71	8.17	57	2.33	2.82	35	1.9%	3.38 [1.06, 5.70]
Cao Z. 2016	12.7	2.6	65	9.2	1.2	56	6.4%	3.50 [2.79, 4.21]
Morell-Garcia D. 2020	6	16	38	2	2	76	0.5%	4.00 [−1.11, 9.11]
Kim J. 2016	14.7	16	251	9.7	12.2	973	2.2%	5.00 [2.88, 7.12]
Liu C.D. 2017	7.9	3.7	31	1.6	0.7	30	4.0%	6.30 [4.97, 7.63]
Pusuroglu H. 2017	49	47	27	36	41	21	0.0%	13.00 [−11.94, 37.94]
Pelaia C. 2021	39	24	116	25	13	40	0.4%	14.00 [8.06, 19.94]
Yardim-Akaydin S. 2014	56.2	37.8	36	33.2	4.9	27	0.1%	23.00 [10.51, 35.49]
**Subtotal (95% CI)**			**1372**			**2170**	**100.0%**	**1.40 [1.04, 1.77]**
Heterogeneity: $Tau^2 = 0.40$; $Chi^2 = 338.92$, df = 23 (P < 0.00001); $I^2 = 93\%$								
Test for overall effect: Z = 7.64 (P < 0.00001)								
**2.2.3 servere OSA VS no OSA**								
Jung J.H. 2021	0.6	0.74	27	0.38	0.33	21	6.5%	0.22 [−0.09, 0.53]
Chen D.D. 2020	1.22	0.34	37	0.81	0.22	17	6.7%	0.41 [0.26, 0.56]
Mônico-Neto M. 2018	3.4	0.4	123	2.9	0.5	211	6.7%	0.50 [0.40, 0.60]
Cofta S. 2009	2.5	1.34	13	2	1.02	14	5.6%	0.50 [−0.40, 1.40]
Chung S. 2007	1.2	1.46	40	0.63	0.83	22	6.2%	0.57 [−0.00, 1.14]
Aihara K. 2011	2.1	3.2	73	1.4	2.7	20	4.7%	0.70 [−0.69, 2.09]
Masood R.K. 2017	1.72	1.47	90	0.63	0.35	63	6.5%	1.09 [0.77, 1.41]
Kapsimalis F. 2008	3.5	3	26	1.9	1	15	4.9%	1.60 [0.34, 2.86]
Tanriverdi H. 2016	4.3	3.05	18	2.05	3.38	24	3.6%	2.25 [0.30, 4.20]
Xie J.Y. 2020	3.14	2.77	59	0.82	1.21	34	5.8%	2.32 [1.50, 3.14]
Sanz-Rubio D. 2020	4	3.4	55	1.3	1.2	31	5.5%	2.70 [1.71, 3.69]
Zhang L. 2020	10.8	5.6	53	8	5.4	19	2.3%	2.80 [−0.06, 5.66]
Saletu M. 2006	5.8	6.5	51	2.8	4.6	44	3.1%	3.00 [0.76, 5.24]
Israel L.P. 2013	4.5	2.1	25	1.5	1	24	5.6%	3.00 [2.08, 3.92]
Bhushan B. 2009	4.4	1.6	26	1.4	1.4	46	6.0%	3.00 [2.26, 3.74]
Uygur F. 2016	4.9	1.8	32	1.4	0.9	31	6.0%	3.50 [2.80, 4.20]
Li F. 2016	6.45	7.25	68	2.33	2.82	35	3.6%	4.12 [2.16, 6.08]
Cao Z. 2016	14.2	3.3	53	9.2	1.2	56	5.6%	5.00 [4.06, 5.94]
Liu C.D. 2017	9.3	4.1	20	1.6	0.7	30	3.8%	7.70 [5.89, 9.51]
Cilekar S. 2021	31.2	31.5	20	21	12.1	40	0.1%	10.20 [−4.11, 24.51]
Pelaia C. 2021	48	38	162	25	13	40	0.5%	23.00 [15.90, 30.10]
Yardim-Akaydin S. 2014	66.6	57.5	67	33.2	4.9	27	0.1%	33.40 [19.51, 47.29]
Pusuroglu H. 2017	76	88	26	36	41	21	0.0%	40.00 [1.90, 78.10]
Balci M. M. 2012	63	25	31	16	7	33	0.3%	47.00 [37.88, 56.12]
**Subtotal (95% CI)**			**1195**			**918**	**100.0%**	**2.48 [1.94, 3.02]**
Heterogeneity: $Tau^2 = 1.15$; $Chi^2 = 521.73$, df = 23 (P < 0.00001); $I^2 = 96\%$								
Test for overall effect: Z = 8.93 (P < 0.00001)								

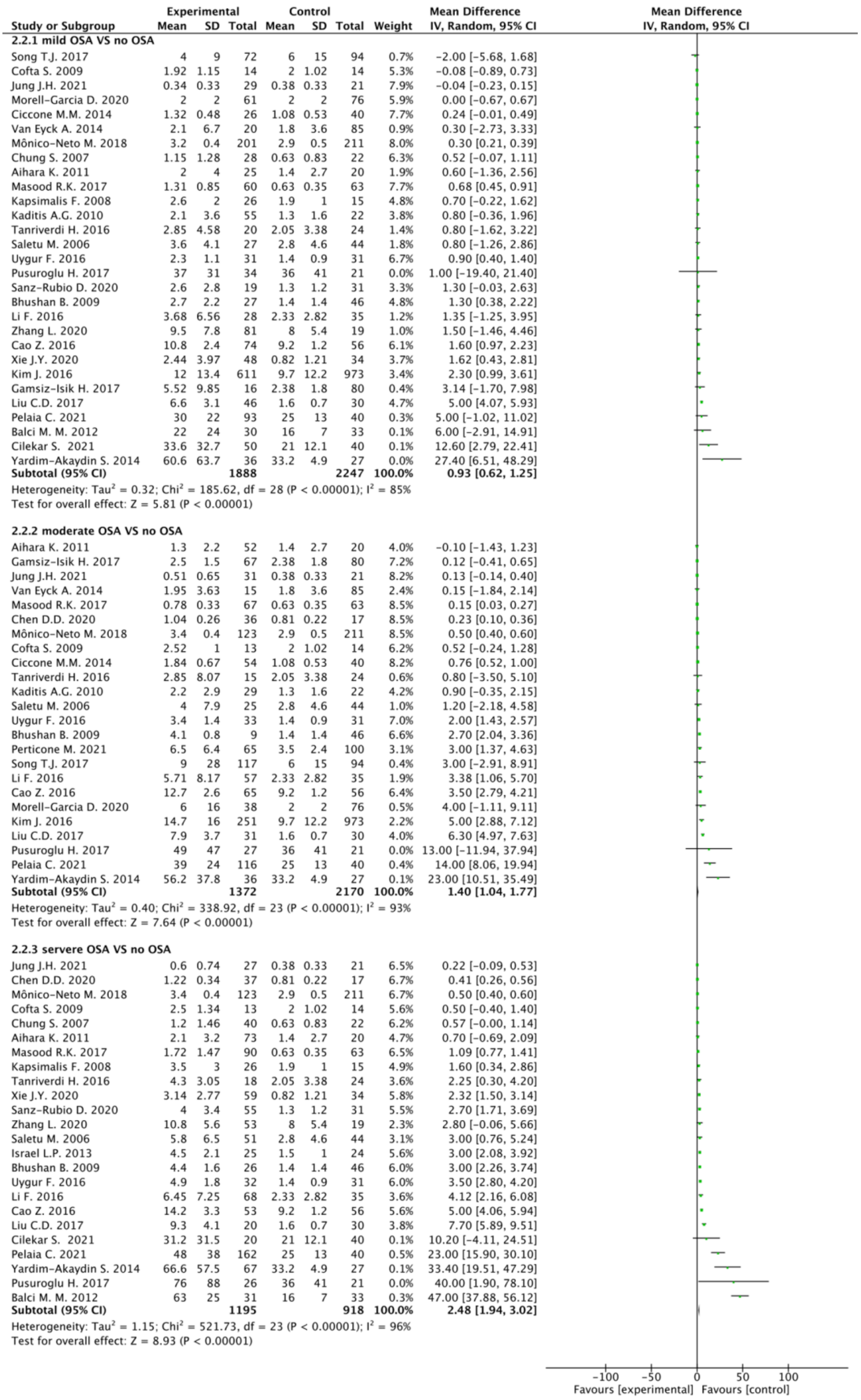

Supplemental figure 4. Forest plot of CRP concentration difference between mild, moderate, severe OSA patients and controls.

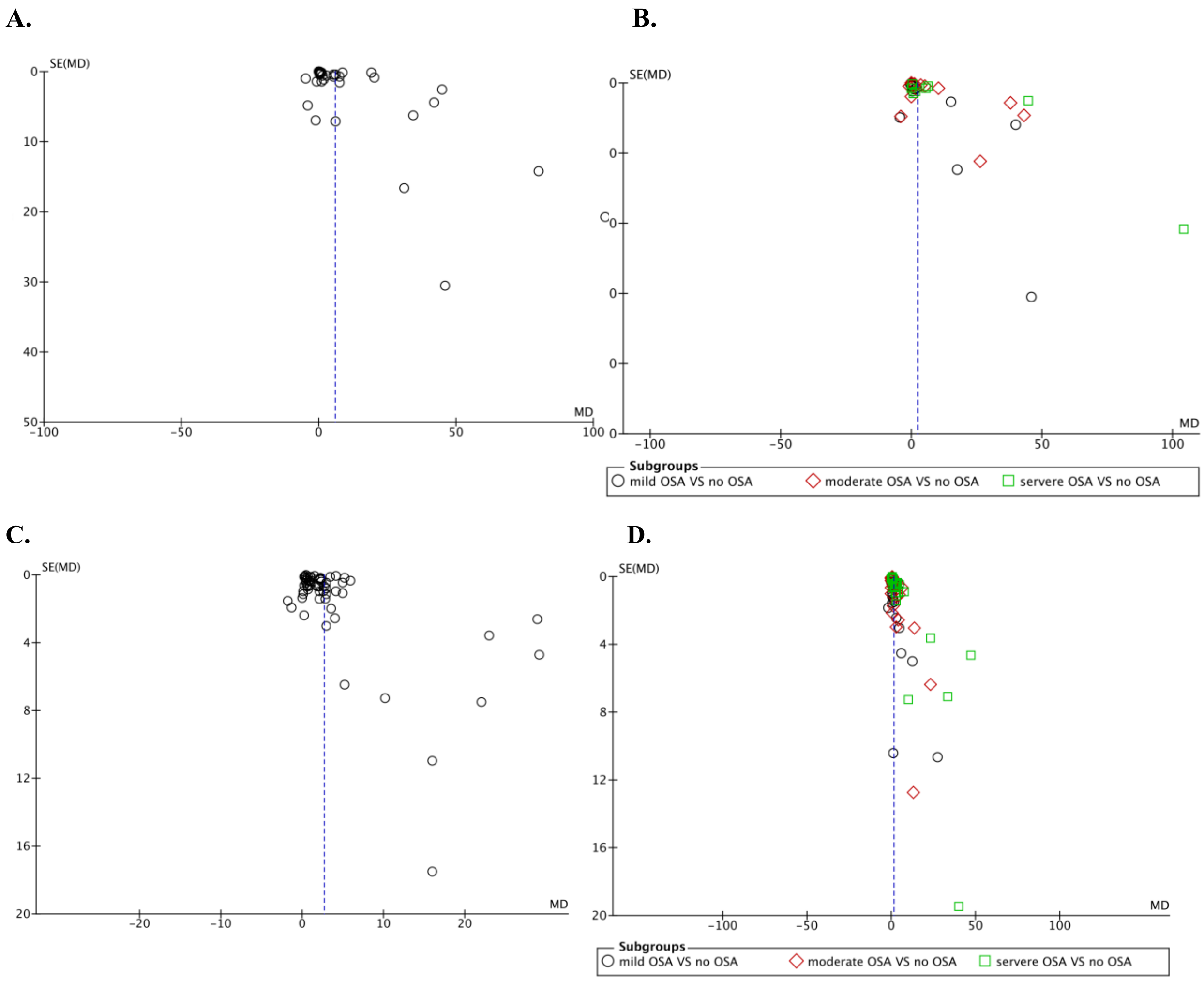

Supplemental figure 5. Funnel plots of concentration differences between OSA patients and controls. (A). TNF-α; (B). TNF-α in mild, moderate, severe OSA; (C) CRP; (D) CRP in mild, moderate, severe OSA.

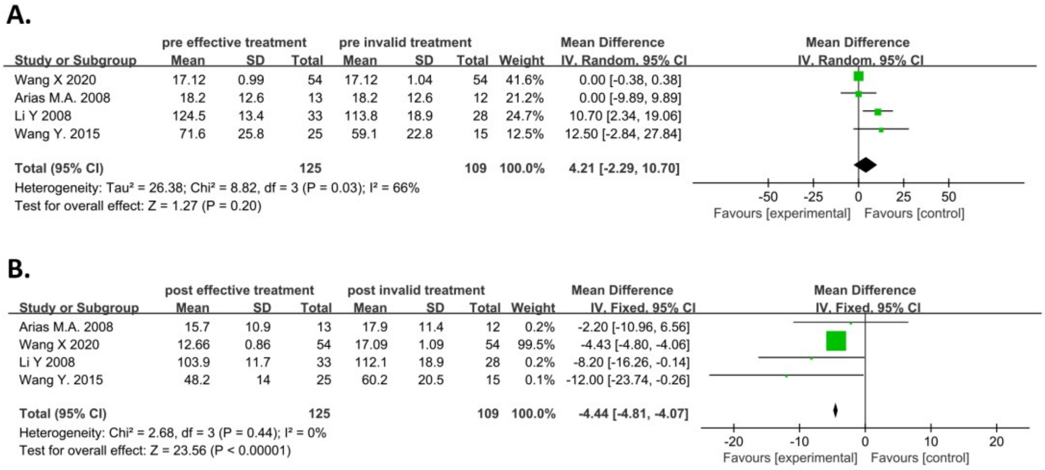

Supplemental figure 6. Forest plots of TNF-α levels in OSA participants between CPAP group and non-CPAP group. (A). baseline comparison between CPAP and non-CPAP groups, (B). post treatment comparison between CPAP and non-CPAP groups.

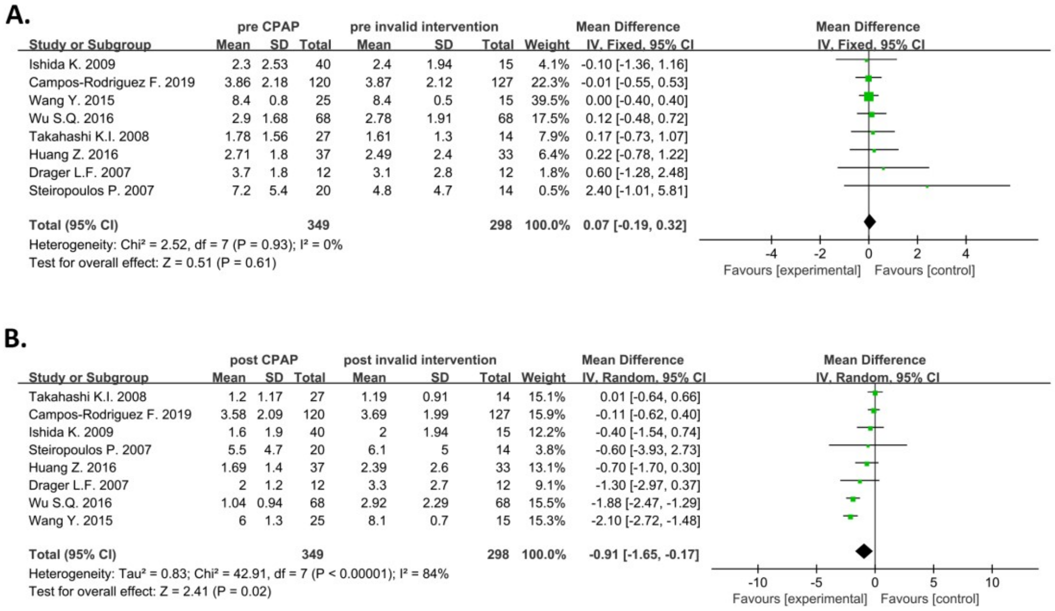

Supplemental figure 7. Forest plots of CRP levels in OSA participants between CPAP group and non-CPAP group. (A). baseline comparison between CPAP and non-CPAP groups, (B). post treatment comparison between CPAP and non-CPAP groups.

A. baseline

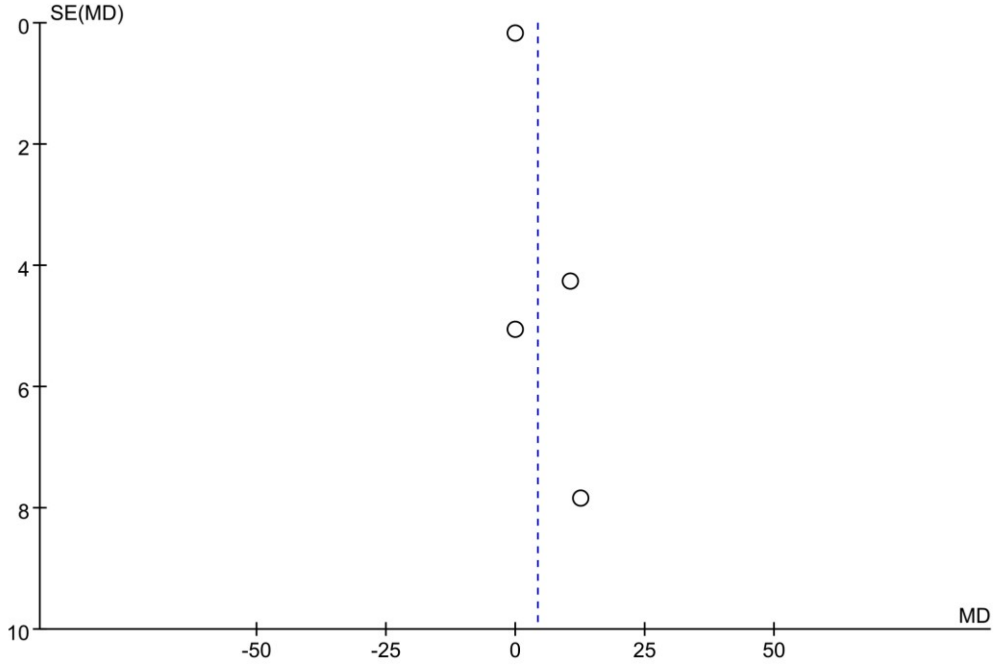

B. post

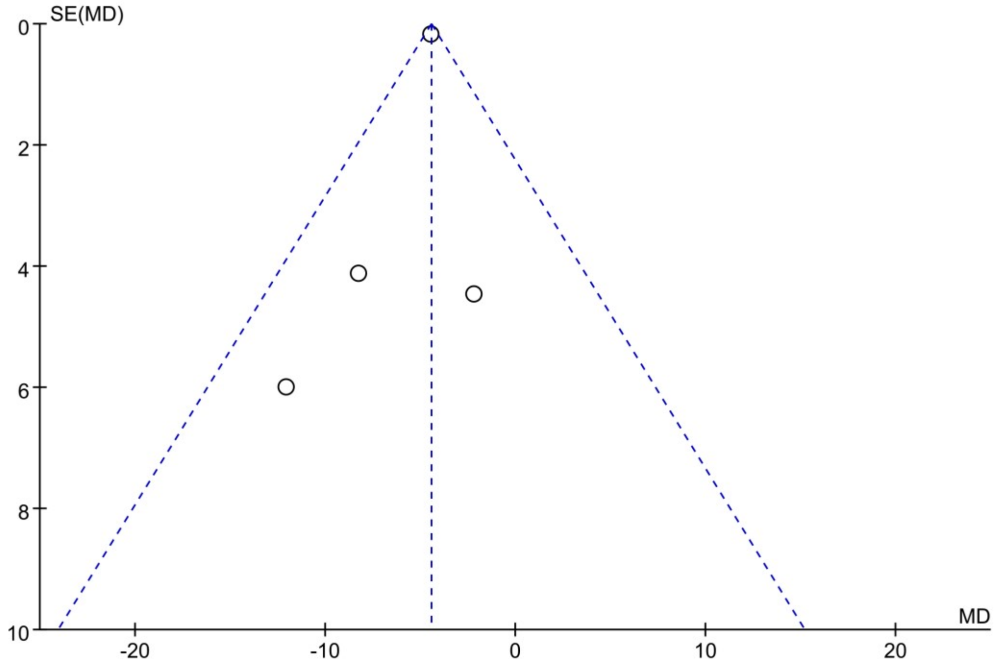

Supplemental figure 8. Funnel plots of TNF-α levels in OSA participants between CPAP group and non-CPAP group. (A). baseline comparison between CPAP and non-CPAP groups, (B). post treatment comparison between CPAP and non-CPAP groups.

A. baseline

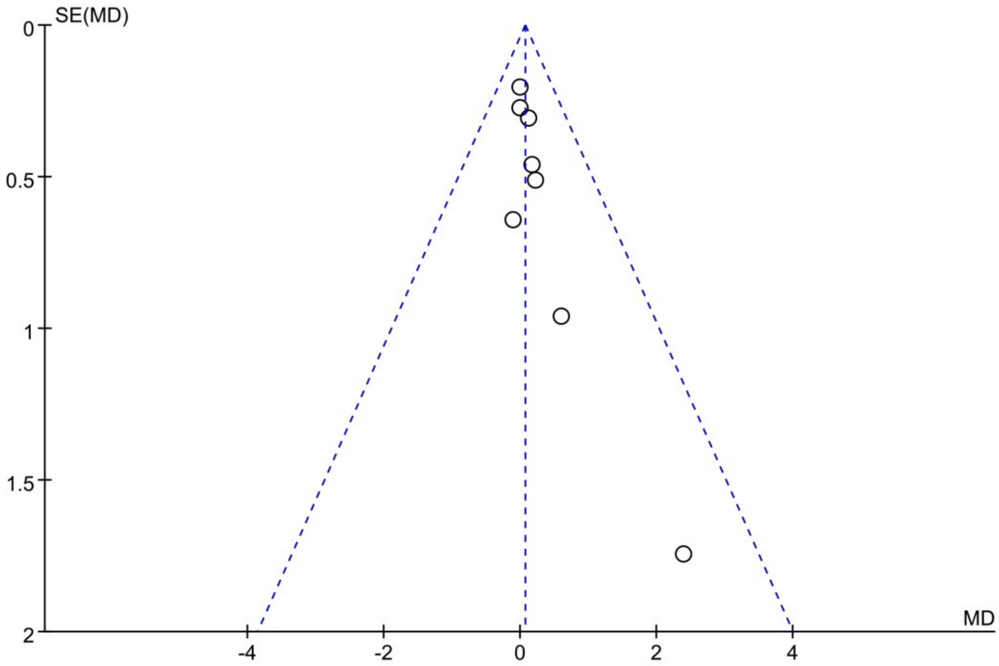

B. post

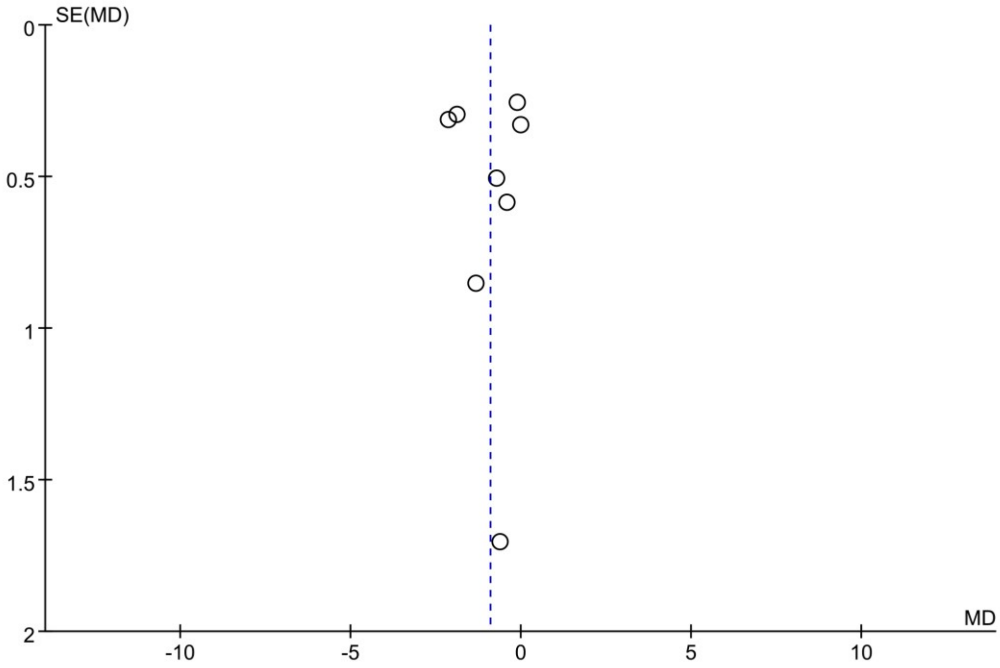

Supplemental figure 9. Funnel plots of CRP levels in OSA participants between CPAP group and non-CPAP group. (A). baseline comparison between CPAP and non-CPAP groups, (B). post treatment comparison between CPAP and non-CPAP groups.
