# Supplementary material for: The causal relationships between obstructive sleep apnea and elevated CRP and TNF-α protein levels
Source: Ann Med. 2022 Jun 2;54(1):1578–89. doi: 10.1080/07853890.2022.2081873 (PMC9176672; doi:10.1080/07853890.2022.2081873)
Supplement: Supplemental Material [file IANN_A_2081873_SM7792.zip › Supplemental files/20220528_Supplementary Tables[AU] (1).pdf]

Supplemental material 2:

Supplemental tables.

Supplemental table 1. Characteristics of the included literature for TNF-α protein level and OSA.

Study ID	Groups	NO.	TNF-α, pg/ml mean ± SD	AHI, events/h	Age, years	BMI, kg/m^2	Sex, M/F	Sample sources	Detection Method
Bilal N. 2021	mild	10	11.75±2.64	9.70±2.77	42.70±14.25	30.65±6.98	5/5	serum	ELISA
	moderate	10	11.94±1.35	20.24±4.40	47.00±9.63	30.66±7.76	8/2		
	severe	10	16.11±2.2	44.01±10.07	43.30±7.42	29.93±3.71	7/3		
	control	30	10.58±1.71	2.62±1.34	42.53±9.81	29.09 ±4.52	14/16		
Ji L. 2021	mild	20	52.5±50.9	11.09±3.04	42.75±14.55	34.07±8.53	12/8	serum	ELISA
	moderate	20	61.2±45.1	23.77±3.68	44.15±15.99	31.19±7.75	13/7		
	severe	27	138.9±105.3	69.51±23.64	45.19±13.84	33.22±9.08	16/11		
	control	21	34.9±21.9	2.69±0.35	42.97±10.26	31.57±8.85	13/8		
Chen V.G. 2020	mixed	17	50.39±25.86	/	3-12	/	/	palatine tonsils	Milliplex-Human Cytokine/Chemokine reading kit
	control	17	44.41±13.77	/	/	/	/		
ChuangH. H. 2020	mixed	11	44.9±19.3	22.3±14.4	7.6±2.5	/	9/2	serum	Bio-Plex® Pro Human Cytokine 27-plex panel
	control	24	45.9±19.2	3.7±2.6	6.8±1.7	/	16/8		
Ming H. 2019	mixed	684	31.2±5.3	31.15±9.12	51.34±5.16	/	446/238	serum	ELISA
	control	192	12.1±1.1	4.34±2.01	52.18±4.51	/	128/64		
Rogers V.E. 2018	mixed	20	4.5±3.18	13.1±9.8	4-12	/	/	serum	Milliplex® Human Cytokine Multiplex Immunoassay kits
	control	7	5.24±3.25	0.8±0.3	4-12	/	/		
Bozic J. 2018	moderate	25	5.79±1.44	21.69±3.92	53.92±10.75	28.42±2.57	25/0	plasma	ECLIA
	severe	25	8.67±2.41	48.26±18.10	52.04±13.11	29.30±2.74	25/0		
	control	25	2.35±1.25	/	52.52±10.18	27.78±2.23	25/0		

Gamsiz-Isik H. 2017	mild	16	11.5±4.6	/	/	/	/	GCF	ELISA
	moderate-severe	67	10.3±2.59	/	/	/	/		
	mixed	83	11.5±3.11	/	46.87±8.21	31.53±3.44	65/18		
	control	80	11.25±4	/	44.23±9.83	30.91 ±3.31	57/23		
Jin F. 2017	mixed	100	37.67±0.21	38.011±8.040	55.284±7.128	26.746±3.500	82/18	plasma	ELISA
	control	50	29.15±1.74	3.623±1.537	56.131±6.210	25.196±2.449	37/13		
Smith D.F. 2017	mild	23	5.9±6	3.5±1.1	9.6±2.5	20.7±4.6	8/15	plasma	human multiplex cytokine assay
	moderate-severe	42	6±4.1	15.2±11.7	9.0±2.7	23.4±13.5	18/24		
	control	90	10.1±45.4	0.4±0.3	9.7±2.5	19.4±4.4	41/49		
Zhang Z. 2017	mixed	50	3.01±0.21	/	79.38 months	/	40/10	serum	flow cytometry
	control	52	2.89±0.23	/	77.13 months	/	33/19		
De Santis S. 2015	mild	9	120±14.1	/	/	29.88±7.4	/	serum	ELISA
	moderate-severe	17	123.4±11.1	/	/	34.64±2.6	/		
	mixed	26	122.2±12	26.15±12.1	41.8±7.4	33.0±5.2	17/9		
	control	24	80.2±18.3	1.65±0.9	43.7±8.2	30.8±4.3	16/8		
Leon-Cabrera S. 2015	mixed	29	337.9±67.8	/	37.2±11.4	45.2±8.4	4/25	serum	ELISA
	control	13	306.9±38.9	/	33.5±10.9	45.4±8.2	2/11		
Ciccone M.M. 2014	mild	26	14.42±3.29	10.55±3.14	53.65±11.47	28.13±3.04	23/3	plasma	ELISA
	moderate-severe	54	22.83±3.85	45.13±16.08	52.33±10.19	28.8±3.03	45/9		
	mixed	80	20.09±5.39	/	/	/	68/12		
	control	40	12.53±3.48	2.11±1.14	52.27±10.52	28.24±2.7	34/6		
Nobili V. 2014	mild	26	6.9±2.1	1.96±0.41	11.8±2.0	28.1±5.1	15/11	serum	ELISA
	severe	13	6.9±2.3	8.71±2.5	11.9±2.7	30.1±5.3	7/6		
	mixed	39	2.2±6.6	4.43±2.89	11.8±2.0	28.3±5.2	22/17		
	control	26	6.8±2	0.51±0.27	11.6±2.0	26.4±5.8	16/10		
Unuvar Dogan F. 2014	moderate-severe	33	0.128±0.15	47.2±23.2	45.3±8.5	31.0±1.7	33/0	serum	ELISA
	control	24	0.108±0.027	3.6±1.8	40.5±9.5	30.7±1.5	24/0		

Akinnusi M. 2013	moderate-severe	25	6.83±2.55	/	/	/	25/0	monocyte	ELISA
	control	18	1.57±0.44	1.9±1.6	52.3±9.3	29.5±5.1	18/0		
Alexopoulos E.I. 2013	mild	22	0.65±0.18	2.1±1.0	6.0±2.5	0.4±1.6, z score	14/8	plasma	ELISA
	moderate-severe	24	0.63±0.2	11.5±5.1	5.7±2	1.1±1.3, z score	13/11		
	control	22	0.63±0.17	0.5±0.3	6.8±2.1	1.1±1.6, z score	12/10		
Yang D. 2013	mixed	25	12.55±8.09	24±17	54±7	27.39±2.91	23/2	plasma	ELISA
	control	25	5.12±1.23	3±1	53±7	26.27±1.9	23/2		
Medeiros C. A. M. 2012	mild-moderate	15	0.84±1.8	/	62.62±9.0	24.50±3.8	11/4	serum	ELISA
	severe	35	2.09±7.3	/	65.0±7.2	25.9±4.1	20/15		
	control	15	0.32±0.77	/	62.50±8.4	25.81±4.04	6/9		
Kim J. 2010	moderate	9	14.56±5.61	14.40±4.07	38±15.04	24.43±2.45	/	plasma	ELISA
	severe	28	15.32±6.8	52.71±22.23	42±10.77	28.69±4.05	/		
	control	22	14.4±4.13	1.25±1.25	26±6.91	23.88±2.30	/		
Sahlman J. 2010	mild	84	1.54±1.75	9.6±2.9	50.4±9.3	32.5±3.3	64/20	plasma	ELISA
	control	40	1.17±1.58	1.9±1.4	45.6±11.5	31.5±3.5	25/15		
Steiropoulos P. 2010	mixed	38	6.72±3.72	61±27	45.5±10.5	36.4±7.4	33/5	blood	quantitative sandwich enzyme immunoassay technique
	control	23	3.94±1.34	5.3±3.2	43.7±6.7	34.5±3.7	17/6		
Li Y. 2009	mild	22	102.3±11.3	14.1±3.5	48±12	25.7±4.2	15/7	serum	ELISA
	moderate	22	125±11.9	29.7±5.5	44±13	28.8±5.3	18/4		
	severe	24	132.1±10.8	70.1±18.1	44±8	28.67±4.2	17/7		
	control	22	87.3±6.1	2.9±1.3	43±93	23.3±2.0	14/8		
Antonopoulou S. 2008	mixed	45	1.4±0.9	39±25	52±12	33.5±7	37/8	EBC	ELISA
	control	25	0.64±0.3	<5	51±7	31±3	18/7		
Constantinidis J. 2008	mild-moderate	13	124.64±96.7	3.4±0.3	/	33.4±1.5	13/0	serum	ELISA
	control	12	78.8±50.1	23.6±3.5	/	34.9±1.8	/		
Kanbay A. 2008	mixed	106	114.15±144.15	40.14±14.30	51.39±10.37	31.06±5.87	62/33	plasma	ELISA
	control	32	34.25±13.1	1.96±1.08	44.79±13.35	28.25±5.49	19/13		

Tomiyama H. 2008	mild	10	1.5±0.3	11±3	49±14	25.1±4.5	10/0	plasma	ELISA
	moderate	12	1.9±0.5	24±5	52±17	25.0±2.0	12/0		
	severe	28	2.2±0.6	62±22	52±11	28.3±4.3	28/0		
	control	15	1.7±0.5	/	53±10	24.3±2.5	15/0		
Chen J. 2007	mixed	100	127.6±37.76	/	/	/	67/33	serum	ELISA
	control	40	93.13±32.14	/	/	/	/		
Kobayashi K. 2006	mixed	35	1.11±0.46	52.26±14.76	51.4±13.1	27.9±3.6	30/5	serum	ELISA
	control	16	0.62±0.44	/	41±13.1	27.4±3.7	13/3		
Ciftci T. U. 2004	mixed	43	4.6±3.39	33.19±24.99	49.6±9.1	31.86±4.11	43/0	serum	ELISA
	control	22	3.29±2.13	1.55±0.96	47.2±10.3	31.03±3.1	22/0		
Minoguchi K. 2004	mild	12	1.8±0.43	9.0±3.8	51.0±14.8	26.1±1.3	12/0	serum	ELISA
	moderate-severe	12	2.34±0.54	59.2±14.7	49.2±11.7	29.1±2.2	12/0		
	control	12	1.12±0.39	2.1±0.9	47.5±11.2	22.3±0.9	12/0		
Alberti A. 2003	mixed	18	26.9±3.45	18.2±15.1	52.7±12.0	26.5±2.2	15/3	plasma	ELISA
	control	20	6.5±1.55	<5	51.3±13.2	22.1± 3.4	14/6		
Liu H. 2000	mixed	22	299.09±43.57	44.02±20.97	47.4±13.6	27.58±3.28	15/7	plasma	ELISA
	control	16	101.88±21.27	4.29±2.16	47.6±14. 7	23. 11±2.96	11/5		

GCF: gingival crevicular fluid; ECLIA: electrochemiluminescence immunoassay; ELISA: enzyme-linked immunosorbent assay.

Supplemental table 2. Detailed bibliography for each included publication for TNF-α.

Bilal N. 2021*	1. Bilal N, Kurutas EB, Orhan I, Bilal B, Doganer A. Evaluation of preoperative and postoperative serum interleukin-6, interleukin-8, tumor necrosis factor alpha and raftlin levels in patients with obstructive sleep apnea. *Sleep and Breathing.* 2021;25(2):819-826.
Ji L. 2021*	2. Ji L, Liu Y, Liu P, et al. Serum periostin and TNF-alpha levels in patients with obstructive sleep apnea-hypopnea syndrome. *Sleep and Breathing.* 2021;25(1):331-337.
Chen V.G. 2020	3. Chen VG, Fonseca V, Amaral JB, et al. Inflammatory markers in palatine tonsils of children with obstructive sleep apnea syndrome. *Braz J Otorhinolaryngol.* 2020;86(1):23-29.
ChuangH. H. 2020	4. Chuang HH, Huang CG, Chuang LP, et al. Relationships among and predictive values of obesity, inflammation markers, and disease severity in pediatric patients with obstructive sleep apnea before and after adenotonsillectomy. *Journal of Clinical Medicine.* 2020;9(2).
Ming H. 2019	5. Ming H, Tian A, Liu B, et al. Inflammatory cytokines tumor necrosis factor-α, interleukin-8 and sleep monitoring in patients with obstructive sleep apnea syndrome. *Exp Ther Med.* 2019;17(3):1766-1770.
Rogers V.E. 2018	6. Rogers VE, Bollinger ME, Tulapurkar ME, et al. Inflammation and asthma control in children with comorbid obstructive sleep apnea. *Pediatr Pulmonol.* 2018;53(9):1200-1207.
Bozic J. 2018*	7. Bozic J, Borovac JA, Galic T, Kurir TT, Supe-Domic D, Dogas Z. Adropin and Inflammation Biomarker Levels in Male Patients With Obstructive Sleep Apnea: A Link With Glucose Metabolism and Sleep Parameters. *J Clin Sleep Med.* 2018;14(7):1109-1118.
Gamsiz-Isik H. 2017	8. Gamsiz-Isik H, Kiyan E, Bingol Z, Baser U, Ademoglu E, Yalcin F. Does Obstructive Sleep Apnea Increase the Risk for Periodontal Disease? A Case-Control Study. *J Periodontol.* 2017;88(5):443-449.
Jin F. 2017	9. Jin F, Liu J, Zhang X, et al. Effect of continuous positive airway pressure therapy on inflammatory cytokines and atherosclerosis in patients with obstructive sleep apnea syndrome. *Mol Med Rep.* 2017;16(5):6334-6339.
Smith D.F. 2017*	10. Smith DF, Hossain MM, Hura A, et al. Inflammatory Milieu and Cardiovascular Homeostasis in Children With Obstructive Sleep Apnea. *Sleep.* 2017;40(4).
Zhang Z. 2017	11. Zhang Z, Wang C. Immune status of children with obstructive sleep apnea/hypopnea syndrome. *Pak J Med Sci.* 2017;33(1):195-199.
De Santis S. 2015	12. De Santis S, Cambi J, Tatti P, Bellussi L, Passali D. Changes in ghrelin, leptin and pro-inflammatory cytokines after therapy in Obstructive Sleep Apnea Syndrome (OSAS) patients. *Otolaryngol Pol.* 2015;69(2):1-8.
Leon-Cabrera S. 2015	13. Leon-Cabrera S, Arana-Lechuga Y, Esqueda-León E, et al. Reduced systemic levels of IL-10 are associated with the severity of obstructive sleep apnea and insulin resistance in morbidly obese humans. *Mediators Inflamm.* 2015;2015:493409.
Ciccone M.M. 2014	14. Ciccone MM, Scicchitano P, Zito A, et al. Correlation between inflammatory markers of atherosclerosis and carotid intima-media thickness in Obstructive Sleep Apnea. *Molecules.* 2014;19(2):1651-1662.
Nobili V. 2014	15. Nobili V, Cutrera R, Liccardo D, et al. Obstructive sleep apnea syndrome affects liver histology and inflammatory cell activation in pediatric nonalcoholic fatty liver disease, regardless of obesity/insulin resistance. *Am J Respir Crit Care Med.* 2014;189(1):66-76.
Unuvar Dogan F. 2014	16. Unüvar Doğan F, Yosunkaya S, Kuzu Okur H, Can U. Relationships between Obstructive Sleep Apnea Syndrome, Continuous Positive Airway Pressure Treatment, and Inflammatory Cytokines. *Sleep Disord.* 2014;2014:518920.
Akinnusi M. 2013	17. Akinnusi M, Jaoude P, Kufel T, El-Solh AA. Toll-like receptor activity in patients with obstructive sleep apnea. *Sleep Breath.* 2013;17(3):1009-1016.

Alexopoulos E.I. 2013*	18. Alexopoulos EI, Theologi V, Malakasioti G, et al. Obstructive Sleep Apnea, Excessive Daytime Sleepiness, and Morning Plasma TNF-alpha Levels in Greek Children. *Sleep.* 2013;36(11):1633-1638.
Yang D. 2013	19. Yang D, Liu Z, Luo Q. Plasma ghrelin and pro-inflammatory markers in patients with obstructive sleep apnea and stable coronary heart disease. *Med Sci Monit.* 2013;19:251-256.
Medeiros C. A. M. 2012*	20. Medeiros CA, de Bruin VM, Andrade GM, Coutinho WM, de Castro-Silva C, de Bruin PF. Obstructive sleep apnea and biomarkers of inflammation in ischemic stroke. *Acta Neurol Scand.* 2012;126(1):17-22.
Kim J. 2010*	21. Kim J, Lee CH, Park CS, Kim BG, Kim SW, Cho JH. Plasma levels of MCP-1 and adiponectin in obstructive sleep apnea syndrome. *Arch Otolaryngol Head Neck Surg.* 2010;136(9):896-899.
Sahlman J. 2010	22. Sahlman J, Miettinen K, Peuhkurinen K, et al. The activation of the inflammatory cytokines in overweight patients with mild obstructive sleep apnoea: Sleep apnea and inflammation. *Journal of Sleep Research.* 2010;19(2):341-348.
Steiropoulos P. 2010	23. Steiropoulos P, Papanas N, Nena E, et al. Inflammatory markers in middle-aged obese subjects: does obstructive sleep apnea syndrome play a role? *Mediators Inflamm.* 2010;2010:675320.
Li Y. 2009*	24. Li Y, Chongsuvivatwong V, Geater A, Liu A. Exhaled breath condensate cytokine level as a diagnostic tool for obstructive sleep apnea syndrome. *Sleep Med.* 2009;10(1):95-103.
Antonopoulou S. 2008	25. Antonopoulou S, Loukides S, Papatheodorou G, Roussos C, Alchanatis M. Airway inflammation in obstructive sleep apnea: is leptin the missing link? *Respir Med.* 2008;102(10):1399-1405.
Constantinidis J. 2008	26. Constantinidis J, Ereliadis S, Angouridakis N, Konstantinidis I, Vital V, Angouridaki C. Cytokine changes after surgical treatment of obstructive sleep apnoea syndrome. *Eur Arch Otorhinolaryngol.* 2008;265(10):1275-1279.
Kanbay A. 2008	27. Kanbay A, Kokturk O, Ciftci TU, Tavil Y, Bukan N. Comparison of serum adiponectin and tumor necrosis factor-alpha levels between patients with and without obstructive sleep apnea syndrome. *Respiration.* 2008;76(3):324-330.
Tomiyama H. 2008*	28. Tomiyama H, Okazaki R, Inoue D, et al. Link between obstructive sleep apnea and increased bone resorption in men. *Osteoporos Int.* 2008;19(8):1185-1192.
Chen J. 2007	29. Chen J, Yang J, Yang WW, Wu H. Analysis of serum IL-6 and TNF-α levels in children with obstructive sleep apnea syndrome. *Chinese Journal of Evidence-Based Medicine.* 2007;7(7):547-549.
Kobayashi K. 2006	30. Kobayashi K, Nishimura Y, Shimada T, et al. Effects of continuous positive airway pressure on soluble CD40 ligand in patients with obstructive sleep apnea syndrome. *Chest.* 2006;129(3):632-637.
Ciftci T. U. 2004	31. Ciftci TU, Kokturk O, Bukan N, Bilgihan A. The relationship between serum cytokine levels with obesity and obstructive sleep apnea syndrome. *Cytokine.* 2004;28(2):87-91.
Minoguchi K. 2004*	32. Minoguchi K, Tazaki T, Yokoe T, et al. Elevated production of tumor necrosis factor-alpha by monocytes in patients with obstructive sleep apnea syndrome. *Chest.* 2004;126(5):1473-1479.
Alberti A. 2003	33. Alberti A, Sarchielli P, Gallinella E, et al. Plasma cytokine levels in patients with obstructive sleep apnea syndrome: a preliminary study. *J Sleep Res.* 2003;12(4):305-311.
Liu H. 2000	34. Liu H, Liu J, Xiong S, Shen G, Zhang Z, Xu Y. The change of interleukin-6 and tumor necrosis factor in patients with obstructive sleep apnea syndrome. *J Tongji Med Univ.* 2000;20(3):200-202.

Supplemental table 3. Characteristics of the included literature for CRP protein levels and OSA.

Study ID	Groups	NO.	CRP, mg/L mean ± SD	AHI, events/h	Age, years	BMI, kg/m2	Sex, M/F	Sample sources	Detection method
Bhatt S. P. 2021	mixed	190	3.7±2.12	>=1	10.71±3.00	27.1±6.53	92/41	serum	/
	control	57	1.5±0.71	< 1	11.87±2.66	27.4±4.88	21/11		
Chen Y. C. 2021	mixed	56	3.64±4.73	66.9±12	43.3±8.6	25.9±2.8	50/6	serum	ELISA
	control	16	1.79±1.11	3.7±2.5	41.1±12.8	25.2±2.9	14/2		
Cignarelli A. 2021	mixed	68	82±72	33.77±20.47	46.4±10.4	42.8±9.7	42/26	blood	/
	control	22	66±71	4.10±2.57	39.9±13.7	39.4±8.0	7/15		
Cilekar S. 2021	mild-moderate	50	33.6±32.7	13.10±5.41	50.66±10.67	30.15±3.64	7/43	/	ELISA
	severe	20	31.2±31.5	38.90±5.61	53.10±5.79	31.89±3.93	1/19		
	control	40	21.0±12.1	2.3±2.5	50.68±2.74	30.28 ± 2.81	7/33		
Jung J.H. 2021	mild	29	0.34±0.33	/	45.3±2.9	26.8±3.4	/	serum	/
	moderate	31	0.51±0.65	/	43.8±3.2	25.7±2.1	/		
	severe	27	0.6±0.74	/	48.2±3.1	26.7±3.1	/		
	control	21	0.38±0.33	/	47.1±2.6	27.6 + 8.1	/		
Pelaia C. 2021	mild	93	30 ± 22	5 ≤ AHI < 15	61.1 ± 9.9	33.1 ± 5.6	60/33	blood	immunoturbidimetric method automated system
	moderate	116	39 ± 24	15 ≤ AHI < 30	61.6 ± 11.1	33.6 ± 6.8	78/38		
	severe	162	48 ± 38	AHI ≥ 30	61.3 ±11	35.7 ± 7.8	141/21		
	control	40	25 ± 13	< 5	61 ± 10.5	29.4 ± 5.6	24/16		
Perticone M. 2021	moderate-severe	65	6.5±6.4	42.2±25.1	56.2±11.0	36.9±6.3	48/17	plasma	automated instrument
	control	100	3.5±2.4	2.3±1.4	54.3±10.9	36.6±3.3	62/38		
Rong W. 2021	mixed	42	7.02±0.34	/	57.32±4.68	25.43±1.76	25/17	serum	ELISA
	control	42	2.88±0.22	/	58.17±3.22	26.15±2.43	23/19		

Wang L.J. 2021	mixed	122	52.81±27	34.2±13.6	68.4±6.7	25.8±3.6	106/16	serum	/
	control	130	23.99±10.91	8.6±4.7	68.9±8.0	24.1±3.1	85/45		
Azar C. 2020	mixed	99	9.3±6.5	/	47.85±11.55	33.59±5.6	67/32	/	/
	control	50	9.3±9.3	/	43.28±12.12	32.08±4.1	29/21		
Brener A. 2020	mixed	14	6.4±2.3	7.6±2.3	34.2±4.1	/	14/0	blood	/
	control	44	4.3±2.2	1.4±1.4	32.6±3.9	/	44/0		
Chen D.D. 2020	moderate	36	1.04±0.26	31.96±10.01	43.44±11.61	25.38±2.05	36/0	serum	BN II nephelometer
	severe	37	1.22±0.34	72.24±15.39	41.92±11.46	26.19±3.41	37/0		
	control	17	0.81±0.22	4.37±2.18	41.76±11.71	25.54±2.11	17/0		
Chien M.Y. 2020	mixed	20	2.3±1.4	48.0±18.9	50.2±5.6	26.05±2.92	20/0	blood	CRP-Latex (II) immunoturbidimetric assay kit
	control	20	1.2±0.6	2.4±1.2	50.4±6.7	25.82±2.76	20/0		
Chu A.A. 2020	mixed	71	5.4±1.1	44.3±17.2	48.0±7.8	26.0±2.4	52/19	serum	latex agglutination immunoassay
	control	31	2.0±0.5	2.6±1.0	47.2±8.6	24.9±2.3	19/12		
Huang Y.S. 2020	mixed	55	3.37±6.04	15.71±22.60	7.67±2.64	/	36/19	serum	ELISA
	control	32	0.42±0.23	0.46±0.28	7.02±0.65	/	21/11		
Morell-Garcia D. 2020	mild	61	2±2	2.4±1.0	7.9±3.7	21.0±7.6	/	serum	Architect c16000 platform
	moderate-severe	38	6±16	14.8±4.3	8.6±3.5	23.5±8.2	/		
	control	76	2±2	0.4±0.3	8.6±3.5	21.8±7.1	/		
Sanz-Rubio D. 2020	mild-moderate	19	2.6±2.8	20.5±6.5	45.3±6.8	30.9±4.6	/	serum	automated immunonephelometry
	severe	55	4±3.4	57.7±19.9	44.1±8.9	32.2±5.2	/		
	control	31	1.3±1.2	2.0±2.9	41.5±8.4	27.2±3.1	/		
Xie J.Y. 2020	mild-moderate	48	2.44±3.97	15.75±7.92	48.21±14.01	26.02±3.39	35/13	blood	ELISA
	severe	59	3.14±2.77	60.62±16.56	48.22±11.73	29.34±4.00	52/7		

	control	34	0.82±1.21	2.23±1.49	34.74±14.02	23.80±4.00	18/16		
Zhang L. 2020	mild-moderate	81	9.5±7.8	14.7±7.1	30.2±7.6	41.3±5.9	24/57	blood	immunoassay analyzer
	severe	53	10.8±5.6	59.1±22.8	32.1±7.8	45.5±6.8	32/21		
	control	19	8±5.4	2.8±1.3	27.8±7.3	38.7±3.5	3/16		
Bauça J.M. 2019	mixed	209	41.34±60.87	34.8±17.5	60.2±10.6	29.33±4.42	168/41	serum	/
	control	152	36.08±61.19	6.2±4.	57.1±11.9	26.75±3.77	132/20		
Bhatt S.P. 2019	mixed	47	3.6 ± 1.5	13.5 ± 6.4	44.2 ± 9.1	32.5 ± 6.9	25/22	serum	/
	control	25	1.4 ± 0.7	2.3 ± 1.1	41 ± 8.5	28.5 ± 8.6	17/8		
Voulgaris A. 2019	mixed	64	5.5±5.8	53.9 ± 28.5	51±12.2	35.9±13.1	54/10	plasma	random-access chemistry analyzer
	control	32	3.4±3.6	2.4 ± 1.3	50.1±11.7	33.9±8.8	22/10		
Aydin S. 2018	mixed	47	42±38	/	42.8±8.4	30.4±3.9	47/0	blood	/
	control	17	20±21	/	39.2±8.7	25.7±2.8	17/0		
Horvath P. 2018	mixed	50	4.2±3.7	22.2(5.7–119.4)	61±9	31±6	27/23	serum	/
	control	26	4±1.8	1.8(0.0–4.8)	56±8	26±3	9/17		
Kunos L. 2018	mixed	45	6.3±13	27.8±21.6	60±11	31.0±6.5	27/18	serum	/
	control	31	2.8±2.4	2.3±1.2	53±15	25.4±3.6	11/21		
Mônico-Neto M. 2018	mild	201	3.2±0.4	10± 0.4	49±12.3	27±0.2	91/110	serum	/
	moderate	123	3.4±0.4	21±0.7	55±12.4	22±0.2	69/54		
	severe	123	3.4±0.4	45±1.3	58±13.4	28±0.2	70/53		
	control	211	2.9±0.5	3±0.3	42±10.4	26±0.2	61/150		
Zhang D.M. 2018	mixed	30	2.09±1.8	61.48±15.00	40.73±8.90	28.85±2.62	30/0	plasma	automated biochemistry instrument
	control	20	1.19±1.14	1.93±1.38	36.10±13.67	27.55±2.97	20/0		

Alonso-Álvarez M.L. 2017	mixed	62	4.71±10.95	/	/	/	28/34	plasma	/
	control	51	2.54±2.60	/	/	/	28/23		
Gamsiz-Isik H. 2017	mixed	83	4.95±8.95	/	46.87±8.21	31.53±3.44	65/18	serum	ELISA
	mild	16	5.52±9.85	/	/	/	/		
	moderate-severe	67	2.5±1.5	/	/	/	/		
	control	80	2.38±1.80	/	44.23±9.83	30.91 ±3.31	57/23		
Jin F. 2017	mixed	100	49.64±21.66	38.011±8.040	55.284±7.128	26.746±3.500	82/18	plasma	ELISA
	control	50	6.37±1.30	3.623±1.537	56.131±6.210	25.196±2.449	37/13		
Liu C.D. 2017	mild	46	6.6±3.1	11.2±3.1	50. 9±10. 1	28. 7±3. 2	38 /8	blood	ELISA
	moderate	31	7.9±3.7	22.9±6.3	51. 6±10. 4	29. 7±3. 3	27 /4		
	severe	20	9.3±4.1	55.3±12.6	51. 8±10. 5	31. 4±3. 5	18 /2		
	mixed	97	7.5±3.5	24.1±17.1	51. 3±10. 3	29. 9±3. 3	83 /14		
	control	30	1.6±0.7	3.1±1.1	50.6±9. 8	27.5±3. 1	26 /4		
Masood R.K. 2017	mild	60	1.31±0.85	/	49.85±2.11	24.03±2.74	46/14	serum	/
	moderate	67	0.78±0.33	/	50.37±2.28	27.00±3.03	60/7		
	severe	90	1.72±1.47	/	52.34±2.60	26.54±2.66	74/16		
	control	63	0.63±0.35	/	45.22±2.57	23.90±2.66	36/27		
Nakabayashi K. 2017	mixed	134	0.96±2.49	/	69.1±11.5	25.4±4.46	95/39	serum	/
	control	109	0.52±1.41	/	69.7±10.2	23.2±3.33	82/27		
Pusuroglu H. 2017	mild	34	37±31	9.7±4.7	51.6±6.5	32.9±4.2	22/12	serum	/
	moderate	27	49±47	25.3±21.5	53.6±6.7	35.5±5.5	20/7		
	severe	26	76±88	56.6±21.6	55±7.5	37.3±6.9	20/6		
	mixed	87	52±59	28.5±25.7	53.1±7	35.1±5.8	62/25		
	control	21	36±41	5.4±3.8	51.8±7.3	35.2±4.7	18/3		

Song T.J. 2017	mild	72	4±9	9.3±2.8	61.7±13.4	25.2±3.0	42/30	serum	/
	moderate-severe	117	9±28	37.3±18.6	63.1±12.9	25.4±3.7	78/39		
	control	94	6±15	1.4±1.5	56.9±13.6	23.5±3.0	38/56		
Xu Q. 2017	mixed	33	1.47±1.6	19.6±4.7	51.6±9.8	30.1±3.5	23/10	serum	IMMULITE 2000 system
	control	30	0.97±1.22	2.2±1.5	49.2±13.1	28.9±4.4	21/9		
Zhang D. 2017	mixed	38	2.19±1.94	58.70(36.63-71.15)	40.87±9.01	28.84±2.45	38/0	plasma	/
	control	10	2.09±3.46	0.55(0.08-1.50)	30.80±10.25	22.39±1.75	10/0		
Cao Z. 2016	mild	74	10.8±2.4	/	52.2±13.6	25.5±3.5	41/33	serum	/
	moderate	65	12.7±2.6	/	53.3±12.2	25.8±4.6	35/30		
	severe	53	14.2±3.3	/	55.3±13.1	26.3±3.9	31/22		
	control	56	9.2±1.2	/	49.4±11.6	24.2±2.7	30/26		
Huang Y.S. 2016	mixed	47	1.90±0.44	9.13±1.67	7.84±0.56	16.95±0.47	30/17	serum	ELISA
	control	32	0.41±0.48	0.37±0.06	7.02±0.65	16.55±0.58	21/11		
Kim J. 2016	mild	611	12±13.4	8.8±2.7	57.2±7.2	25.1±2.6	348/263	serum	ADVIA 1650 and 1680
	moderate-severe	251	14.7±16	24.7±10.8	58.0±7.7	25.9±3.2	167 /84		
	control	973	9.7±12.2	1.9±1.4	53.8±6.6	23.9±2.6	453/520		
Li F. 2016	mild	28	3.68±6.56	/	41±12	26.07±4.11	28/0	serum	ELISA
	moderate	57	5.71±8.17	/	45±8	26.80±2.54	57/0		
	severe	68	6.45±7.25	/	45±7	27.15±2.20	68/0		
	control	35	2.33±2.82	/	43±9	25.62±2.79	35/0		
Tanriverdi H. 2016	mild	20	2.85±4.58	/	/	/	/	venous blood	/
	moderate	15	2.85±8.07	/	/	/	/		
	severe	18	4.3±3.05	/	/	/	/		

	mixed	53	2.8±3.8	27.5±22.7	49.9±8.8	31.6±5.2	45/8		
	control	24	2.05±3.38	1.73±1.2	44.2±13.4	49.9±8.8	21/3		
Uygur F. 2016	mild	31	2.3±1.1	/	/	/	/	serum	immunoturbidimetric assay
	moderate	33	3.4±1.4	/	/	/	/		
	severe	32	4.9±1.8	/	/	/	/		
	mixed	96	3.6±1.8	/	51.4±9.7	30.8±3.7	66/30		
	control	31	1.4±0.9	/	50.6±12.8	29.6±4.1	23/8		
Zhang H. 2016	mixed	41	4.25±0.61	37.55±4.62	48.08±7.14	24.77±1.51	41/0	serum	/
	control	19	3.32±0.35	3.65±0.42	47.45±8.37	24.48±1.66	19/0		
Wang Y. 2015	mixed	47	8.4±0.7	34.0(24.4-43.7)	60.5±5.2	31.4±2.1	43/4	serum	/
	control	28	8.1±0.8	1.7(0.8-2.7)	58.6±5.9	30.4±2.1	26/2		
Ciccone M.M. 2014	mild	26	1.32±0.48	10.55±3.14	53.65±11.47	28.13±3.04	23/3	serum	latex-particle enhanced turbidimetric immunoassay
	moderate-severe	54	1.84±0.67	45.13±16.08	52.33±10.19	28.8±3.03	45/9		
	control	40	1.08±0.53	2.11±1.14	52.27±10.52	28.24±2.7	34/6		
Fouda N. 2014	mixed	14	6.7±0.6	/	41.2±5.11	30.8±2.48	0/14	blood	atex-particle enhanced turbidimetric immunoassay
	control	16	4.9±0.3	/	39.9±6.6	20.3±1.55	0/16		
Van Eyck A. 2014	mild	20	2.1±6.7	3.4±0.9,OAHI	11±3	30.0±6.4	9/11	serum	high-sensitivity immune-turbidimetric test
	moderate-severe	15	1.95±3.63	11.2±7.9,OAHI	12±3	31.9±5.9	10/5		
	control	85	1.8±3.6	0.4±0.9,OAHI	12±3	30.6±5.7	30/55		
Yardim-Akaydin S. 2014	mild	36	60.6±63.7	/	/	/	21/15	serum	nephelometer
	moderate	36	56.2±37.8	/	/	/	24/12		
	severe	67	66.6±57.5	/	/	/	49/18		

	mixed	139	62.3±54.7	male:38.0±26.1; female:29.5±24.3	/	/	94/45		
	control	27	33.2±4.9	male:2.8±1.2; female:2.7±1.5	/	/	14/13		
Bezerra P.C 2013	mixed	27	6.7±5	/	52.41±9.44	36.82±7.44	0/27	serum	/
	control	21	8±7.9	/	/	/	0/21		
Israel L.P. 2013	severe	25	4.5±2.1	14.1±2.9	5.1±3.2	/	11/14	serum	particle-enhanced immunonephelometry
	control	24	1.5±1	0.6±0.2	5.3±3.5	/	11/13		
Nural S. 2013	mixed	25	8.35±9.5	58.59±24.76	58.60±10.93	34.79±7.63	25/0	serum	Immage 800® (USA) device
	control	25	8.12±7.2	2.78±1.66	61.24±8.56	26.73±4.32	25/0		
Balci M. M. 2012	mild-moderate	30	22±24	14.2±14.6	42.5±11.2	26.9±2.4	18/12	serum	enzyme-linked immunosorbent assay
	severe	31	63±25	66.3±39.9	45.7±10.3	27.3±2.3	16/15		
	control	33	16±7	3.2 ±1.9	41.6±11.6	26.3±1.4	16/17		
Feng X. 2012	mixed	132	3.84±1.25	/	47.51±10.31	27.17±3.77	132/0	serum	auto biochemistry instrument
	control	108	2.76±0.91	/	47.29±10.89	27.07±3.10	108/0		
Aihara K. 2011	mild	25	2±4	9.8±2.8	56.7±15.7	25.7±4.1	16/9	serum	ELISA
	moderate	52	1.3±2.2	22.1±4.3	57.5±15.6	25.6±5.3	37/15		
	severe	73	2.1±3.2	54.3±21.6	57.3±13.5	28.7±6.1	62/11		
	control	20	1.4±2.7	2.2±1.5	43.6±17.7	24.8±3.4	12/8		
Guasti L. 2011	mixed	16	2.98±2.7	AHI >20	61±10	31.76±4.39	15/1	serum	/
	control	11	4.81±4.72	AHI <5	55±14	27.68±3.46	7/4		
	mild	55	2.1±3.6	2.4±1.1, OAHI	6.4±2.6	/	35/20	plasma	/

Kaditis A.G. 2010	moderate-severe	29	2.2±2.9	13.9±13.0, OAHI	5.4±1.5	/	20/9		
	control	22	1.3±1.6	0.6±0.2, OAHI	6.8±2.6	/	11/11		
Bhushan B. 2009	mild	27	2.7±2.2	/	44.9±11.9	30.3±4.8	/	serum	ELISA
	moderate	9	4.1±0.8	/	40.4±11.5	29.7±3.8	/		
	severe	26	4.4±1.6	/	43.8±10.5	31.9±4.2	/		
	mixed	62	3.6±2	/	43.8±11.2	30.9±4.4	53/9		
	control	46	1.4±1.4	/	41.7±6.9	29.9±3.0	30/16		
Cofta S. 2009	mild	14	1.92±1.15	7.85±2.5	50±12	29.3±5.4	14/0	serum	/
	moderate	13	2.52±1	17.8±9.5	51±8	30.4±3.8	13/0		
	severe	13	2.5±1.34	51.5±12.6	51±11	30.7±3.5	13/0		
	control	14	2±1.02	2.2±1.2	50±10	30.2±5.4	14/0		
Kapsimalis F. 2008	mild-moderate	26	2.6±2	16.2±5.2	50.5±13.8	29.4±3.8	26/0	serum	latex particle-enhanced turbidimetric immunoassay
	severe	26	3.5±3	48.1±15.6	55.3±11.6	30.6±3.4	26/0		
	control	15	1.9±1.0	3.1±1.1	47.0±12.5	28.7±4.3	15/0		
Takahashi K.I. 2008	mixed	41	1.72±1.47	/	49.8±10.0	29.4±4.20	38/3	serum	nephelometry
	control	12	0.87±0.96	/	46.7±11.2	25.7±4.10	11/1		
Chung S. 2007	mild-moderate	28	1.15±1.28	/	41.9±9.6	26.3±4.1	28/0	serum	turbidimetric immunoassay
	severe	40	1.2±1.46	/	43.3±8.8	26.6±2.6	40/0		
	control	22	0.63±0.83	/	42.1±8.7	26.2±3.9	22/0		
Saletu M. 2006	mild	27	3.6±4.1	9.6±3.0	55±12	28±5	/	blood	/
	moderate	25	4±7.9	21.1±4.3	55±10	29±5	/		
	severe	51	5.8±6.5	55.5±24.5	54±11	33±5	/		
	control	44	2.8±4.6	1.9±1.3	50±14	27±6	/		
	mixed	146	4.64±6.74	/	46.81±11.42	29.39±7.05	105/41	serum	nephelometry

Guilleminault C. 2004	control	54	4.10±2.1	/	43.87±9.79	24.74±5.34	31/23

ELISA: enzyme-linked immunosorbent assay.

Supplemental table 4. Detailed bibliography for each included publication for CRP.

Bhatt S. P. 2021	1. Bhatt SP, Guleria R, Kabra SK. Metabolic alterations and systemic inflammation in overweight/obese children with obstructive sleep apnea. *PLoS One.* 2021;16(6):e0252353.
Chen Y. C. 2021	2. Chen YC, Hsu PY, Chin CH, et al. H3K23/H3K36 hypoacetylation and HDAC1 up-regulation are associated with adverse consequences in obstructive sleep apnea patients. *Sci Rep.* 2021;11(1):20697.
Cignarelli A. 2021	3. Cignarelli A, Ciavarella A, Barbaro M, et al. Postprandial glucose and HbA1c are associated with severity of obstructive sleep apnoea in non-diabetic obese subjects. *J Endocrinol Invest.* 2021;44(12):2741-2748.
Cilekar S. 2021	4. Cilekar S, Beysel S, Karatas S, Balci A, Akaslan K, Uncu A. Circulating sTweak is associated with visceral adiposity and severity in patients with obstructive sleep apnea syndrome. *Sci Rep.* 2021;11(1):22058.
Jung J.H. 2021	5. Jung JH, Park JW, Kim DH, Kim ST. The Effects of Obstructive Sleep Apnea on Risk factors for Cardiovascular diseases. *Ear Nose Throat J.* 2021;100(5_suppl):477s-482s.
Pelaia C. 2021	6. Pelaia C, Armentaro G, Miceli S, et al. Association Between Sleep Apnea and Valvular Heart Diseases. *Front Med (Lausanne).* 2021;8:667522.
Perticone M. 2021	7. Perticone M, Maio R, Scarpino PE, et al. Continuous Positive Airway Pressure Improves Renal Function in Obese Patients With Obstructive Sleep Apnea Syndrome. *Front Med (Lausanne).* 2021;8:642086.
Rong W. 2021	8. Rong W, Yan Y, Wang A, et al. CHANGES in INFLAMMATORY RESPONSE, ABNORMAL BLOOD COAGULATION and HEMORHEOLOGY in PATIENTS with ISCHEMIC STROKE with OBSTRUCTIVE SLEEP APNEA SYNDROME and THEIR CORRELATIONS. *Acta Medica Mediterranea.* 2021;37(1):293-297.
Wang L.J. 2021	9. Wang LJ, Pan LN, Yan RY, Quan WW, Xu ZH. Obstructive sleep apnea increases heart rhythm disorders and worsens subsequent outcomes in elderly patients with subacute myocardial infarction. *J Geriatr Cardiol.* 2021;18(1):30-38.
Azar C. 2020	10. Azar C, Abakay Ö, Azar Ş, Kermenli T, Yüksel H. The relationship between the heart-type fatty acid binding protein levels, carotid intima media thickness and epicardial fat thickness in patients with obstructive sleep apnea syndrome. *Tuberkuloz ve Toraks.* 2020;68(1):1-8.
Brener A. 2020	11. Brener A, Lebenthal Y, Levy S, Dunietz GL, Sever O, Tauman R. Mild maternal sleep-disordered breathing during pregnancy and offspring growth and adiposity in the first 3 years of life. *Sci Rep.* 2020;10(1):13979.
Chen D.D. 2020	12. Chen DD, Huang JF, Huang SP, Chen XF. Association of serum adiponectin level with cystatin C in male patients with obstructive sleep apnea syndrome. *Sleep Breath.* 2020;24(3):953-960.
Chien M.Y. 2020	13. Chien MY, Lee PL, Yu CW, Wei SY, Shih TT. Intramyocellular Lipids, Insulin Resistance, and Functional Performance in Patients with Severe Obstructive Sleep Apnea. *Nat Sci Sleep.* 2020;12:69-78.
Chu A.A. 2020	14. Chu AA, Yu HM, Yang H, et al. Evaluation of right ventricular performance and impact of continuous positive airway pressure therapy in patients with obstructive sleep apnea living at high altitude. *Sci Rep.* 2020;10(1):20186.
Huang Y.S. 2020	15. Huang YS, Chin WC, Guilleminault C, Chu KC, Lin CH, Li HY. Inflammatory Factors: Nonobese Pediatric Obstructive Sleep Apnea and Adenotonsillectomy. *J Clin Med.* 2020;9(4).
Morell-Garcia D. 2020	16. Morell-Garcia D, Toledo-Pons N, Sanchis P, et al. Red cell distribution width: a new tool for the severity prediction of sleep apnoea syndrome in children. *ERJ Open Res.* 2020;6(4).

Sanz-Rubio D. 2020	17. Sanz-Rubio D, Sanz A, Varona L, et al. Forkhead Box P3 Methylation and Expression in Men with Obstructive Sleep Apnea. *Int J Mol Sci.* 2020;21(6).
Xie J.Y. 2020	18. Xie JY, Liu WX, Ji L, et al. Relationship between inflammatory factors and arrhythmia and heart rate variability in OSAS patients. *Eur Rev Med Pharmacol Sci.* 2020;24(4):2037-2053.
Zhang L. 2020	19. Zhang L, Zhang X, Meng H, Li Y, Han T, Wang C. Obstructive sleep apnea and liver injury in severely obese patients with nonalcoholic fatty liver disease. *Sleep Breath.* 2020;24(4):1515-1521.
Bauça J.M. 2019	20. Bauça JM, Barcelo A, Fueyo L, et al. Biomarker panel in sleep apnea patients after an acute coronary event. *Clin Biochem.* 2019;68:24-29.
Bhatt S.P. 2019	21. Bhatt SP, Guleria R, Vikram NK, Gupta AK. Non-alcoholic fatty liver disease is an independent risk factor for inflammation in obstructive sleep apnea syndrome in obese Asian Indians. *Sleep Breath.* 2019;23(1):171-178.
Voulgaris A. 2019	22. Voulgaris A, Archontogeorgis K, Nena E, et al. Serum levels of NGAL and cystatin C as markers of early kidney dysfunction in patients with obstructive sleep apnea syndrome. *Sleep Breath.* 2019;23(1):161-169.
Aydin S. 2018	23. Aydin Ş, Özdemir C, Küçükali CI, et al. Reduced Peripheral Blood Mononuclear Cell ROCK1 and ROCK2 Levels in Obstructive Sleep Apnea Syndrome. *In Vivo.* 2018;32(2):319-325.
Horvath P. 2018	24. Horvath P, Tarnoki DL, Tarnoki AD, et al. Complement system activation in obstructive sleep apnea. *J Sleep Res.* 2018;27(6):e12674.
Kunos L. 2018	25. Kunos L, Horvath P, Kis A, et al. Circulating Survivin Levels in Obstructive Sleep Apnoea. *Lung.* 2018;196(4):417-424.
Mônico-Neto M. 2018	26. Mônico-Neto M, Moreira Antunes HK, Dos Santos RVT, et al. Physical activity as a moderator for obstructive sleep apnoea and cardiometabolic risk in the EPISONO study. *Eur Respir J.* 2018;52(4).
Zhang D.M. 2018	27. Zhang DM, Pang XL, Huang R, Gong FY, Zhong X, Xiao Y. Adiponectin, Omentin, Ghrelin, and Visfatin Levels in Obese Patients with Severe Obstructive Sleep Apnea. *Biomed Res Int.* 2018;2018:3410135.
Alonso-Álvarez M.L. 2017	28. Alonso-Álvarez ML, Terán-Santos J, Gonzalez Martinez M, et al. Metabolic biomarkers in community obese children: effect of obstructive sleep apnea and its treatment. *Sleep Med.* 2017;37:1-9.
Gamsiz-Isik H. 2017	29. Gamsiz-Isik H, Kiyan E, Bingol Z, Baser U, Ademoglu E, Yalcin F. Does Obstructive Sleep Apnea Increase the Risk for Periodontal Disease? A Case-Control Study. *J Periodontol.* 2017;88(5):443-449.
Jin F. 2017	30. Jin F, Liu J, Zhang X, et al. Effect of continuous positive airway pressure therapy on inflammatory cytokines and atherosclerosis in patients with obstructive sleep apnea syndrome. *Mol Med Rep.* 2017;16(5):6334-6339.
Liu C.D. 2017	31. Liu CD, Wang YS, Jiang Y, Wang SK. Application of Ischemia Modified Albumin in the Diagnosis and Treatment of Obstructive Sleep Apnea Syndrome. *Chinese Journal of Pharmaceutical Biotechnology.* 2017;24(5):409-413.
Masood R.K. 2017	32. Masood RK, Shoaib M, Falak S. Relationship of platelet-lymphocyte ratio with severity of obstructive sleep apnea syndrome. *Medical Forum Monthly.* 2017;28(11):110-114.
Nakabayashi K. 2017	33. Nakabayashi K, Jujo K, Saito K, Oka T, Hagiwara N. Evaluation of the association between sleep apnea and polyunsaturated fatty acids profiles in patients after percutaneous coronary intervention. *Heart Vessels.* 2017;32(11):1296-1303.
Pusuroglu H. 2017	34. Pusuroglu H, Somuncu U, Bolat I, et al. Galectin-3 is associated with coronary plaque burden and obstructive sleep apnoea syndrome severity. *Kardiol Pol.* 2017;75(4):351-359.

Song T.J. 2017	35. Song TJ, Park JH, Choi KH, et al. Is obstructive sleep apnea associated with the presence of intracranial cerebral atherosclerosis? *Sleep and Breathing.* 2017;21(3):639-646.
Xu Q. 2017	36. Xu Q, Du J, Ling X, Lu Y. Evaluation of MIh Scoring System in Diagnosis of Obstructive Sleep Apnea Syndrome. *Med Sci Monit.* 2017;23:4715-4722.
Zhang D. 2017	37. Zhang DM, Huang R, Xiao Y, Gong FY, Zhong X, Luo JM. Secreted Frizzled-Related Protein 5 (SFRP5) in Patients with Obstructive Sleep Apnea. *Chin Med Sci J.* 2017;32(4):211-217.
Cao Z. 2016	38. Cao Z, Zhang P, He Z, et al. Obstructive sleep apnea combined dyslipidemia render additive effect on increasing atherosclerotic cardiovascular diseases prevalence. *Lipids Health Dis.* 2016;15:98.
Huang Y.S. 2016	39. Huang YS, Guilleminault C, Hwang FM, et al. Inflammatory cytokines in pediatric obstructive sleep apnea. *Medicine (Baltimore).* 2016;95(41):e4944.
Kim J. 2016	40. Kim J, Lee SJ, Choi KM, et al. Obstructive Sleep Apnea Is Associated with Elevated High Sensitivity C-Reactive Protein Levels Independent of Obesity: Korean Genome and Epidemiology Study. *PLoS One.* 2016;11(9):e0163017.
Li F. 2016	41. Li F, Huang H, Song L, Hao H, Ying M. Effects of Obstructive Sleep Apnea Hypopnea Syndrome on Blood Pressure and C-Reactive Protein in Male Hypertension Patients. *J Clin Med Res.* 2016;8(3):220-224.
Tanriverdi H. 2016	42. Tanriverdi H, Uygur F, Can M, et al. Serum midkine levels in patients with obstructive sleep apnoea. *Acta Medica Mediterranea.* 2016;32(1):127-133.
Uygur F. 2016	43. Uygur F, Tanrıverdi H, Can M, et al. Association between continuous positive airway pressure and circulating omentin levels in patients with obstructive sleep apnoea. *Sleep Breath.* 2016;20(3):939-945.
Zhang H. 2016	44. Zhang H, Feng L, Wu X, Niu Y, Du H. Prognostic role of advanced glycation end products in male patients with obstructive sleep apnoea syndrome and hypertension. *International Journal of Clinical and Experimental Medicine.* 2016;9(7):13135-13141.
Wang Y. 2015	45. Wang Y, Hu K, Liu K, et al. Obstructive sleep apnea exacerbates airway inflammation in patients with chronic obstructive pulmonary disease. *Sleep Med.* 2015;16(9):1123-1130.
Ciccone M.M. 2014	46. Ciccone MM, Scicchitano P, Zito A, et al. Correlation between inflammatory markers of atherosclerosis and carotid intima-media thickness in Obstructive Sleep Apnea. *Molecules.* 2014;19(2):1651-1662.
Fouda N. 2014	47. Fouda N, Dayem AA. Obstructive sleep apnea in patients with rheumatoid arthritis: Correlation with disease activity and pulmonary function tests. *Egyptian Rheumatologist.* 2014;36(4):165-171.
Van Eyck A. 2014	48. Van Eyck A, Van Hoorenbeeck K, De Winter BY, et al. Sleep-disordered breathing and C-reactive protein in obese children and adolescents. *Sleep Breath.* 2014;18(2):335-340.
Yardim-Akaydin S. 2014	49. Yardim-Akaydin S, Caliskan-Can E, Firat H, Ardic S, Simsek B. Influence of gender on C-reactive protein, fibrinogen, and erythrocyte sedimentation rate in obstructive sleep apnea. *Antiinflamm Antiallergy Agents Med Chem.* 2014;13(1):56-63.
Bezerra P.C 2013	50. Bezerra PC, do Prado M, Gaio E, Franco OL, Tavares P. The use of dual-energy X-ray absorptiometry in the evaluation of obesity in women with obstructive sleep apnea-hypopnea syndrome. *Eur Arch Otorhinolaryngol.* 2013;270(4):1539-1545.
Israel L.P. 2013	51. Israel LP, Benharoch D, Gopas J, Goldbart AD. A pro-inflammatory role for nuclear factor kappa B in childhood obstructive sleep apnea syndrome. *Sleep.* 2013;36(12):1947-1955.
Nural S. 2013	52. Nural S, Günay E, Halici B, Celik S, Ünlü M. Inflammatory processes and effects of continuous positive airway pressure (CPAP) in overlap syndrome. *Inflammation.* 2013;36(1):66-74.

Balci M. M. 2012	53. Balci MM, Arslan U, Firat H, et al. Serum levels of adipocyte fatty acid-binding protein are independently associated with left ventricular mass and myocardial performance index in obstructive sleep apnea syndrome. *J Investig Med.* 2012;60(7):1020-1026.
Feng X. 2012	54. Feng X, Li P, Zhou C, Jia X, Kang J. Elevated levels of serum chemerin in patients with obstructive sleep apnea syndrome. *Biomarkers.* 2012;17(3):248-253.
Aihara K. 2011	55. Aihara K, Oga T, Harada Y, et al. Comparison of biomarkers of subclinical lung injury in obstructive sleep apnea. *Respir Med.* 2011;105(6):939-945.
Guasti L. 2011	56. Guasti L, Marino F, Cosentino M, et al. Cytokine production from peripheral blood mononuclear cells and polymorphonuclear leukocytes in patients studied for suspected obstructive sleep apnea. *Sleep and Breathing.* 2011;15(1):3-11.
Kaditis A.G. 2010	57. Kaditis AG, Alexopoulos EI, Karathanasi A, et al. Adiposity and low-grade systemic inflammation modulate matrix metalloproteinase-9 levels in Greek children with sleep apnea. *Pediatr Pulmonol.* 2010;45(7):693-699.
Bhushan B. 2009	58. Bhushan B, Guleria R, Misra A, Pandey RM, Luthra K, Vikram NK. Obstructive sleep apnoea correlates with C-reactive protein in obese Asian Indians. *Nutr Metab Cardiovasc Dis.* 2009;19(3):184-189.
Cofta S. 2009	59. Cofta S, Wysocka E, Michalak S, Piorunek T, Batura-Gabryel H, Torlinski L. Endothelium-derived markers and antioxidant status in the blood of obstructive sleep apnea males. *Eur J Med Res.* 2009;14 Suppl 4(Suppl 4):49-52.
Kapsimalis F. 2008	60. Kapsimalis F, Varouchakis G, Manousaki A, et al. Association of sleep apnea severity and obesity with insulin resistance, C-reactive protein, and leptin levels in male patients with obstructive sleep apnea. *Lung.* 2008;186(4):209-217.
Takahashi K.I. 2008	61. Takahashi K, Chin K, Nakamura H, et al. Plasma thioredoxin, a novel oxidative stress marker, in patients with obstructive sleep apnea before and after nasal continuous positive airway pressure. *Antioxid Redox Signal.* 2008;10(4):715-726.
Chung S. 2007	62. Chung S, Yoon IY, Shin YK, et al. Endothelial dysfunction and C-reactive protein in relation with the severity of obstructive sleep apnea syndrome. *Sleep.* 2007;30(8):997-1001.
Saletu M. 2006	63. Saletu M, Nosiska D, Kapfhammer G, et al. Structural and serum surrogate markers of cerebrovascular disease in obstructive sleep apnea (OSA) - Association of mild OSA with early atherosclerosis. *Journal of Neurology.* 2006;253(6):746-752.
Guilleminault C. 2004	64. Guilleminault C, Kirisoglu C, Ohayon MM. C-reactive protein and sleep-disordered breathing. *Sleep.* 2004;27(8):1507-1511.

Supplemental table 5. **Detailed bibliography for each included publication for TNF-α and CRP levels in OSA participants with interventions of CPAP and non-CPAP.**

Wang X 2020	1. Wang X, Yue Z, Liu Z, et al. Continuous positive airway pressure effectively ameliorates arrhythmias in patients with obstructive sleep apnea-hypopnea via counteracting the inflammation. *Am J Otolaryngol.* 2020;41(6):102655.
Wang Y. 2015(1)	2. Wang Y, Hu K, Liu K, et al. Obstructive sleep apnea exacerbates airway inflammation in patients with chronic obstructive pulmonary disease. *Sleep Med.* 2015;16(9):1123-1130.
Arias M.A. 2008	3. Arias MA, García-Río F, Alonso-Fernández A, et al. CPAP decreases plasma levels of soluble tumour necrosis factor-α receptor 1 in obstructive sleep apnoea. *European Respiratory Journal.* 2008;32(4):1009-1015.
Li Y 2008	4. Li Y, Chongsuvivatwong V, Geater A, Liu A. Are biomarker levels a good follow-up tool for evaluating obstructive sleep apnea syndrome treatments? *Respiration.* 2008;76(3):317-323.
Campos-Rodriguez F. 2019	5. Campos-Rodriguez F, Asensio-Cruz MI, Cordero-Guevara J, et al. Effect of continuous positive airway pressure on inflammatory, antioxidant, and depression biomarkers in women with obstructive sleep apnea: a randomized controlled trial. *Sleep.* 2019;42(10).
Huang Z. 2016	6. Huang Z, Liu Z, Zhao Z, Zhao Q, Luo Q, Tang Y. Effects of Continuous Positive Airway Pressure on Lipidaemia and High-sensitivity C-reactive Protein Levels in Non-obese Patients with Coronary Artery Disease and Obstructive Sleep Apnoea. Heart Lung Circ. 2016;25(6):576-583.
Wu S.Q. 2016	7. Wu SQ, Liao QC, Xu XX, Sun L, Wang J, Chen R. Effect of CPAP therapy on C-reactive protein and cognitive impairment in patients with obstructive sleep apnea hypopnea syndrome. *Sleep Breath.* 2016;20(4):1185-1192.
Ishida K. 2009	8. Ishida K, Kato M, Kato Y, et al. Appropriate use of nasal continuous positive airway pressure decreases elevated C-reactive protein in patients with obstructive sleep apnea. *Chest.* 2009;136(1):125-129.
Takahashi K.I. 2008	9. Takahashi K, Chin K, Nakamura H, et al. Plasma thioredoxin, a novel oxidative stress marker, in patients with obstructive sleep apnea before and after nasal continuous positive airway pressure. *Antioxid Redox Signal.* 2008;10(4):715-726.
Drager L.F. 2007	10. Drager LF, Bortolotto LA, Figueiredo AC, Krieger EM, Lorenzi GF. Effects of continuous positive airway pressure on early signs of atherosclerosis in obstructive sleep apnea. *Am J Respir Crit Care Med.* 2007;176(7):706-712.
Steiropoulos P. 2007	11. Steiropoulos P, Tsara V, Nena E, et al. Effect of continuous positive airway pressure treatment on serum cardiovascular risk factors in patients with obstructive sleep apnea-hypopnea syndrome. *Chest.* 2007;132(3):843-851.
